# MicroCT analysis of connectivity in porous structures: optimizing data acquisition and analytical methods in the context of tissue engineering

**DOI:** 10.1098/rsif.2019.0833

**Published:** 2020-04-22

**Authors:** Malavika Nair, Jennifer H. Shepherd, Serena M. Best, Ruth E. Cameron

**Affiliations:** 1 Cambridge Centre for Medical Materials, Department of Materials Science and Metallurgy, University of Cambridge, 27 Charles Babbage Road, Cambridge CB3 0FS, UK; 2 School of Engineering, University of Leicester, University Road, Leicester LE1 7RH, UK

**Keywords:** MicroCT, percolation, connectivity, porous networks

## Abstract

Micro-computed X-ray tomography (MicroCT) is one of the most powerful techniques available for the three-dimensional characterization of complex multi-phase or porous microarchitectures. The imaging and analysis of porous networks are of particular interest in tissue engineering due to the ability to predict various large-scale cellular phenomena through the micro-scale characterization of the structure. However, optimizing the parameters for MicroCT data capture and analyses requires a careful balance of feature resolution and computational constraints while ensuring that a structurally representative section is imaged and analysed. In this work, artificial datasets were used to evaluate the validity of current analytical methods by considering the effect of noise and pixel size arising from the data capture, and intrinsic structural anisotropy and heterogeneity. A novel ‘segmented percolation method’ was developed to exclude the effect of anomalous, non-representative features within the datasets, allowing for scale-invariant structural parameters to be obtained consistently and without manual intervention for the first time. Finally, an in-depth assessment of the imaging and analytical procedures are presented by considering percolation events such as micro-particle filtration and cell sieving within the context of tissue engineering. Along with the novel guidelines established for general pixel size selection for MicroCT, we also report our determination of 3 μm as the definitive pixel size for use in analysing connectivity for tissue engineering applications.

## 1. Introduction

A key desire in tissue engineering is to mimic existing native environments in order to effectively regenerate them. A single piece of natural tissue exhibits significant heterogeneity in structure, chemistry and biology and thus there has been a drive for increased complexity within scaffold design. Several chemical and biological phenomena, including nutrient diffusion and migration of cells are heavily dependent on the pore size and connectivity. Even though general rules may exist to understand the interdependence between pore size and processing conditions [1] scaffolds require significant structural characterization and analysis.

Micro-computed tomography (MicroCT) provides an obvious tool for the characterization of tissue-engineering scaffolds and development in image analysis, particularly pioneered by Ashworth *et al.* [2–4], has allowed for its application as a predictive tool for cell migration. However, the results from any MicroCT analysis should be treated with a degree of caution. As Liu *et al.* described in 2011 [5] ‘the limitation of microtomography lies in the relationship of the length scale and resolution of the images’ if a higher resolution is desired then typically a smaller length scale will be scanned. This limitation can be extended further, at high resolution a ‘representative volume element’ [5] may consist of a dataset that is too large to be processed for percolation analyses given the constraints of computation placed by commercially available software, and scanning time. On the other hand, if too low a resolution is selected then crucial features may be lost. While the scientific literature generally reports the pixel size for any analysis within the methods sections, it has not been considered as a potential constraint in the case of analysis of pore size or measures of interconnectivity.

Structurally graduated collagen scaffolds were produced in recent work as a bone marrow analogue within a bio-reactor set-up. Although other bone cells are present *in vivo*, this study was set up such that only the bone marrow-derived megakaryocytes were seeded onto the scaffold, which offered sieving capability and shear flow over the megakaryocyte surfaces to enhance platelet output [6]. Although highly interconnected systems have been produced by Haugen *et al.* [7] and Tresoldi *et al.* [8], the pore structure was desired such that the larger megakaryocytes (approx. 30 μm in size) were distributed through the scaffold and only platelets released into the outlet flow. Theoretical analyses of porosity using MicroCT analysis and synthetic micro-particle filtration were applied as predictive tools to tailor cell distribution within and release out of the scaffold. In this study, interconnectivity analysis from MicroCT analysis predicted a highly interconnected structure (over 90% connectivity for theoretical spheres of up to 30 μm diameter) suggesting much reduced sieving capability than was observed experimentally. Thus, there is a need for an in-depth investigation and improvement of current tools available for the measurement of interconnectivity in porous structures.

In this work, we develop guidelines and optimize methods for MicroCT data acquisition and analysis in three stages. Firstly, well-defined artificial microCT datasets are produced to understand the effect of pixel size, feature size, noise and anisotropy using controlled structures devoid of experimental artefacts. We explore ways in which the issues associated with percolation diameter extraction may be overcome through the ‘segmented percolation analysis’ method whereby the ROI is subdivided and analysed post data acquisition. Secondly, after optimization on the artificial datasets, we take structurally variable collagen scaffolds produced by a multi-stage lyophilization process [6] and scan the same volume at a variety of pixel sizes through the application of camera binning. The influence of this pixel size on pore size analysis, interconnectivity and percolation analysis is considered in the two structurally distinct regions of the scaffold using the novel segmented percolation methodology developed in this work. Finally, we provide general guidelines for pixel size selection prior to data capture in tissue-engineering scaffolds. Through the judicious selection of pixel size and the use of the segmented percolation analysis method, this work provides the framework to obtain a value for the percolation diameter consistent with the experimentally observed percolation of micro-particles.

## 2. Material and methods

### 2.1. Terminology

**Figure 1.**
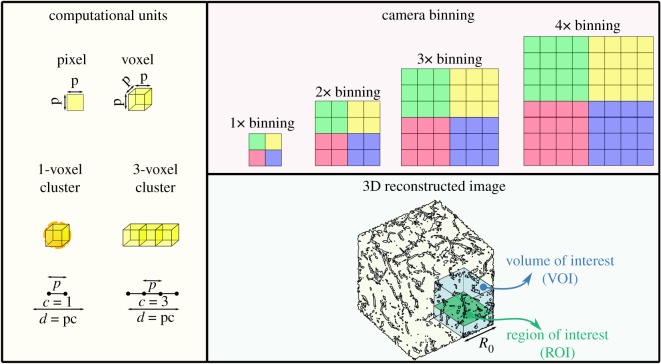
A summary of the terminology used in this paper. A pixel is a 2D picture element that can resolve a physical object of size *p* in one dimension. Pixels can be binned to behave as a singular unit to improve signal-to-noise ratios but at the cost of resolution. A voxel is a 3D computational volume element which can be dimensionalized to a real object using the pixel size *p*. Computational operations may involve the use of clusters of voxels whose size is determined by the number of voxels in one dimension. A volume of interest represents the ‘representative volume element’ chosen by the user for analysis from the macroscopic dataset, whereas a region of interest refers to a 2D slice within each volume of interest.

Table 1 contains the full list of nomenclatures and figure 1 represents a graphical summary of the terminology used in this paper.

**Table 1.** List of nomenclature used for the terminology used in this paper. Where possible, terms are grouped to represent the objects of similar nature such as computational units (*p*, *c*), probes (*d*) and structural elements (*D*).

term	nomenclature
pixel size	*p*
maximum pixel size given resolution constraints	*p*_max_
minimum pixel size given computation constraints	*p*_min_
pixel size constraint given analysis	*p*_analysis_
pixel size constraint given application	*p*_experiment_
voxel cluster size	*c*
maximum voxel cluster size given resolution constraints	*c*_max_
minimum voxel cluster size given computation constraints	*c*_min_
maximum voxel cluster size given resolution and regression constraints	*c*_max,data_
minimum voxel cluster size given computation and regression constraints	*c*_min,data_
voxel cluster diameter	*d*
median interconnection diameter	*d*_median_
percolation diameter	*d*_perc_
percolation diameter stable to ROI subdivision	*d*_perc,stable_
size of a real percolating object of interest	*d*_object_
smallest probe size possessed by virtual percolation object of interest	*d*_probe_
typical protrusion size of a real compressible object	*d*_protrusion_
artificial mesh repeat distance	*D*_repeat_
pore size	*D*_pore_
maximum pore size in a given sample	$D_{\mathrm{pore}}^{\max}$
minimum pore size in a given sample	$D_{\mathrm{pore}}^{\min}$
minimum connection (fenestration) size	*D*_fen_
ROI side length	*R*
original ROI side length	*R*_0_
ROI size at the *i*th iteration of the ROI subdivision method	*R*_*i*_
volume of VOI	*V*
volume of solid material	*V*_*m*_
volume measured after shrink-wrap operation at cluster size *c*	*V*_*c*_
standard deviation in pore sizes	*σ*_pore_
typical length scale of structural variation	*L*_0_
length of accessible pore volume at a voxel cluster size, *c*	*L*_*c*_
critical exponent for percolation analysis	*ν*
granularity in voxel cluster sizes	*g*_*c*_
granularity in ROI subdivision	*g*_sub_
number of data points needed to calculate percolation diameter	*n*_data_

A *pixel* is a 2D picture element and a component of the MicroCT camera whose side size, *p*, determines the ultimate resolution of the scan. For optimum resolution, each pixel of the detector can be treated individually, although the resulting signals are low, leading to long scan times. By applying camera binning, intensities are increased and exposure times reduced. For instance, in 2× camera binning, a pixel size becomes 2 × 2 pixels and with 4×, camera binning is 4 × 4 pixels. Camera binning is not only associated with reduced scan times but also reduced file sizes, thus leading to more efficient image processing.

A *voxel* is a three-dimensional volume element used in the computational rendering of the reconstructed scaffold, which can be dimensionalized to a real object using the pixel size of *p*. Computational operations may involve the use of spherical *voxel clusters* whose size, *c*, is determined by the number of voxels along a diameter. Thus, a *2-voxel cluster* refers to a cluster with a length of 2 voxels along its diameter, with *c* = 2. The *physical diameter of a voxel cluster* can, therefore, be denoted by *d*, where

d=pc.

Finally, the *volume of interest* (VOI) with magnitude *V*, refers to the section of the macroscopic dataset that is chosen by the user as a representative volume element of the larger dataset. A region of interest (ROI), of side length *R*, refers to a selected 2D slice within the volume which may be used for further computational operations.

### 2.2. Artificial mesh generation

**Figure 2.**
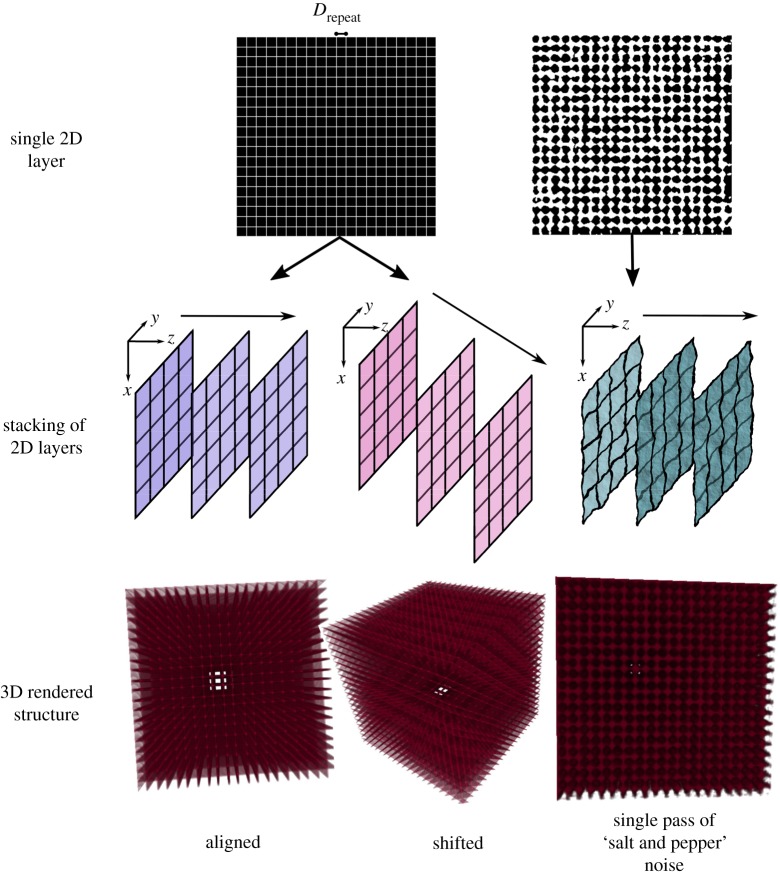
Representative thresholded 2D slices and the 3D volume reconstructions of the artificial meshes. Volumes illustrated here were generated in ImageJ at a simulated pixel size of 6 μm with a grid repeated distance, *D*_repeat_ of 100 μm. All volumes are oriented to indicate the open channels that run through the structure. The black spaces in the 2D slices represent the empty space whereas the white represents struts.

Aligned and shifted artificial meshes were generated in ImageJ [9] (NIH, USA) at simulated pixel sizes of 1.5 μm, 3 μm and 6 μm. To replicate a VOI that is roughly 2 mm^3^, 1333, 666 and 333 images were produced in a given stack for 1.5 μm, 3 μm and 6 μm, respectively. Black and white square meshes as seen in figure 2 were generated in ImageJ with a repeat distance, *D*_repeat_ of 40, 60, 80, 100 and 120 μm within each simulated pixel size, such that ‘black’ pixels represent the empty space and ‘white’ pixels represent the pore struts. Shifted lattices were produced by shifting the origin of the axis by a distance equal to the pixel size in each subsequent slice. The strut thickness in all the shifted and aligned lattices was 1-pixel.

Further lattices were generated from the aligned meshes through noise incorporation. This noise was applied in ImageJ through a sequence of three steps. Firstly, a filter to smooth the active image was applied, whereby each pixel was replaced by the average of its immediate neighbours in the stack. Then, a ‘salt and pepper’ function in ImageJ was applied to the dataset whereby 2.5% of black pixels and white pixels were randomly selected and inverted. Finally, a Gaussian blur filter with a radius of decay equal to two times the stack depth (666 for 6 μm, 1332 for 3 μm and 2666 for 1.5 μm), was applied to smooth the VOI. One, two, four and eight passes of this noise incorporation process were performed on the aligned datasets at a mesh repeat distance of 100 μm at all pixel sizes. Using the CTAnalyzer software (Bruker, Belgium), all datasets were then thresholded and segmented using the automatic Otsu algorithm. Representative 3D volumes to illustrate the creation of aligned, shifted and noisy datasets from 2D image slices are illustrated in figure 2.

### 2.3. Pore size analysis

*Pore sizes* were determined using the 3D object analyser tool within the CTAnalyzer software (Bruker, Belgium). The data were plotted as the mean pore size ± s.d. of the pore size distribution.

### 2.4. Interconnectivity analysis

In order to measure the *interconnectivity*, ROIs were subjected to a shrink-wrap operation. Briefly, this operation uses a *spherical voxel cluster* as a probe to identify accessible pore spaces within the VOI. The ‘3D ROI shrink-wrap’ was performed using the CTAnalyzer software (Bruker, Belgium) with clusters 2–100 voxels in diameter. The 3D object analyser tool in CTAnalyzer (Bruker, Belgium) was used to determine the volume of the simulated struts after each shrink-wrap operation at a given voxel cluster size.

Adopting the expression for interconnectivity from Fostad *et al.* [10], the percentage interconnectivity (I) at given voxel cluster size *c* can be expressed as

$$I=\frac{V-V_{c}}{V-V_{m}}\times 100\text{\%},$$

where *V* is the total VOI, *V*_*m*_ is the volume of the solid material and *V*_*c*_ is the volume measured after a shrink-wrap operation at voxel cluster size *c*.

**Figure 3.**
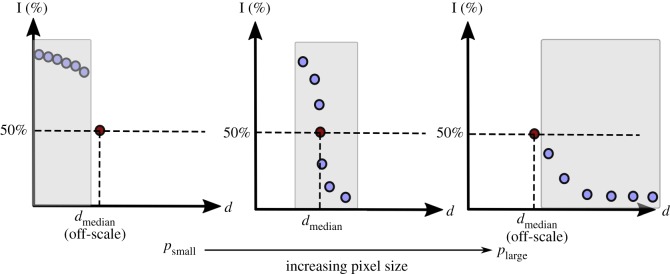
A schematic of the typical interconnectivity plots and median interconnection diameters, *d*_median_, obtained at different pixel sizes. *d*_median_ is defined as the voxel cluster diameter at which only 50% of the scaffold is accessible. Since *d* = *cp*, and the set of cluster sizes available for computation is the same regardless of pixel size, there is a scale accessible by voxel clusters at each pixel size varies (as shown in with the grey boxes). This may result in the median value to be off the accessible scale, and may not be determinable for samples imaged at either too high or too low a pixel size.

Figure 3 illustrates typical interconnectivity plots as well as the *median interconnection diameter*, *d*_median_, the voxel cluster diameter at which only 50% of the scaffold is accessible. *d*_median_ was calculated using a linear interpolation for each interconnectivity measurement where sufficient data points exist to interpolate the value at 50%.

### 2.5. Percolation analysis

The *percolation diameter*, *d*_perc_ may be defined as the diameter of the largest sphere able to penetrate through an infinitely large scaffold and, unlike percentage interconnectivity, is a scalable measure. By increasing the pixel normalized voxel cluster diameter, *d*, the corresponding *length of accessible pore volume* *L* in the *z* direction can be measured. These data can be plotted using the critical scaling law relationship from percolation theory [3] in order to calculate the percolation diameter

$$L=L_{0}{\left(d-d_{\mathrm{perc}}\right)}^{-\nu },$$

where the constants *ν* and *L*_0_ represent the critical scaling exponent, and typical length scale of structural variation, respectively [11].

**Figure 4.**
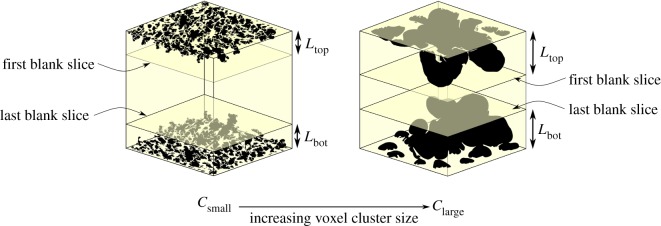
The shrink-wrap process produces stacks of data at each voxel cluster size, *c*, representing open, interconnected pore clusters as black voxels and both struts and other isolated, unconnected clusters as white voxels. The Python script identifies the first and last slices at which no connected pores exist in the stack, represented by an entirely blank slice. The distance from the top of the VOI to the first blank slice, *L*_top_ and the equivalent distance from the bottom, *L*_bot_ were then used to determine the percolation diameter.

Using CTAnalyzer software (Bruker, Belgium), the generated meshes were thresholded and segmented using the automatic Otsu algorithm. A ‘3D ROI shrink-wrap analysis’ was performed in CTAnalyzer using spherical voxel clusters 2–100 voxels in diameter for each dataset, and the resultant ROIs were saved as binarised images. A bespoke Python script was written to analyse the binarised images. As illustrated in figure 4, the script identifies the first and last slice in the stack of images post-shrink-wrap to present entirely blank pixels. A completely blank slice represents a slice unconnected to any pores from either vertical surface. The distance from the surface to the first blank slice in the direction of percolation is then calculated as the percolation depth *L* at that given voxel cluster size.

**Figure 5.**
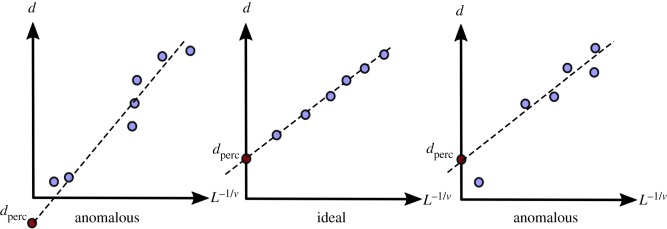
A schematic of the typical percolation linear regression plots. *d*_perc_ is obtained from the intercept of the line drawn to fit the data points obtained from the analysis. The limits of scalability or presence of inhomogeneity may produce anomalous data points, resulting in a deviation from linearity or an unphysical (negative) percolation diameter.

As illustrated in figure 5, the percolation diameter, *d*_perc_, for each dataset was then obtained by using linear regression on the set of *d* and their corresponding *L*^−1/*ν*^, where *ν* = 0.88 for 3D datasets [12].

### 2.6. Segmented percolation analysis

**Figure 6.**
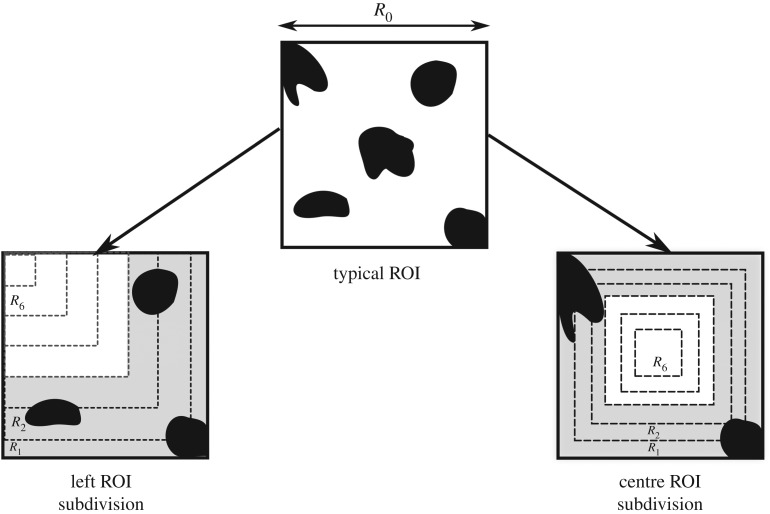
An illustration of the influence of anomalous pathways in the chosen ROI, preventing the calculation of a percolation diameter. The subdivision method (here with *g*_sub_ = 6), calculates a percolation diameter outside the regions (shaded in grey) where such features exist. From the typical ROI shown, anomalous pores which are not in the top right corner of the ROI will be eliminated using the left subdivision method. Similarly, anomalous pores that are not in the centre of the ROI will be eliminated by the centre subdivision method.

The Python script for percolation length calculation was extended to allow for iterative, segmented analysis of the saved binarised images. Using a granularity factor *g*_sub_, the percolation analysis was performed on a subdivided region of the original image such that each ROI is smaller than the previous region analysed by a factor of 1/*g*. This *g*_sub_ represents the number of smaller sections that will be created through each subdivision method. Therefore, at iteration *i*, the dimensions of the image are

$$R_{i}=R_{0}-i\frac{R_{0}}{g_{\mathrm{sub}}},$$

where *R*_*i*_ is the current dimension and *R*_0_ is the original ROI dimension. As seen in figure 6, sets of subdivided images of dimensions *R*_*i*_ × *R*_*i*_ were produced during the analysis. The first set was partitioned such that all subdivided images *R*_*i*_ × *R*_*i*_ share the centre of the original image *R*_0_ × *R*_0_, whereas the second set was designed for each *R*_*i*_ × *R*_*i*_ section to share the top left corner with the original image.

For this analysis, *g*_sub_ was chosen to be 20 for computational convenience.

### 2.7. Percolation diameter selection

Since the subdivision algorithm described in §2.6 produces a percolation diameter for each segmented ROI, a sequence of steps were employed to select a single percolation diameter to characterize the entire dataset. Firstly, all the percolation diameters were rounded to the nearest multiple of the pixel size, in order to reflect the intrinsic limits of resolution. Secondly, all non-positive values were removed from the datasets since they do not represent a physical percolation diameter. Finally, the percolation diameter exhibiting the longest plateau (the most stable, repeated value) with respect to the subdivided region size (ROI subsize) was selected for the ‘left’ subdivision method and for the ‘centre’ subdivision method. Data were plotted as the mean ± s.d. of two percolation diameters (measured from the top and bottom of the stacks as indicated in figure 4) for each mesh generated.

### 2.8. Scaffold fabrication

MicroCT analysis was carried out on structurally variable collagen scaffolds previously produced for the purpose of cell filtration during *ex vivo* platelet generation. These scaffolds were produced by a two-stage lyophilization process as described elsewhere [6,13]. A continuous interface existed between two structurally distinct regions, a top region with a larger more anisotropic structure and a base layer exhibiting a more isotropic, smaller pore structure.

### 2.9. Micro-computed tomography

As previously described [6] 5 mm diameter cylindrical punched samples were analysed using a Skyscan 1272 (Bruker, Belgium) desktop MicroCT system. An initial scan pixel size of 1.5 μm was selected (no applied camera binning), with an operating voltage of 25 kV. These data have been previously published [6] but scanning was then repeated of the same samples with 2× and 4× camera binning applied, resulting in pixel sizes of 3 and 6 μm.

Resulting projections were reconstructed in NRecon (Bruker, Belgium) and systematic VOIs selected as described previously [6]. A three-dimensional analysis was carried out in after automatic Otsu thresholding and sweep despeckling in CTAnalyzer.

### 2.10. VOI selection

For porosity analysis, multiple VOIs of 1 × 1 mm cross-section were selected in the top and bottom regions of the scaffold. The precise thickness of these VOIs was dependent upon the specific structure of each scaffold (ensuring the analysis was performed on the bulk region of the layers and not the interface) but was approximately 1 mm.

### 2.11. Structural analysis

In order to mimic the conditions of cell seeding from a single face, VOIs were initially modified to allow penetration only from the top *x*–*y* plane by inserting a solid border around the ROI. An interconnectivity analysis was carried out using the ‘3D ROI shrink-wrap analysis’ with increasing voxel cluster size using clusters of 2–30 voxels; 3D object analysis was used to calculate the inaccessible volume after each shrink-wrap operation as defined in §2.4. The length of accessible pore volume was calculated manually for these real datasets. After shrink-wrap, the resulting ROIs were saved as stacks of binarised images. For modified ROIs, the distance from the surface to the first entirely blank slice was determined. VOIs were also considered without modification where penetration from all surfaces occurred. The first non-connected slice was defined as the first to be absent of pore necks (i.e. where only surface pores were accessible). The percolation diameter, *d*_perc_, for each dataset was then obtained as described for the artificial meshes before the ROI subdivision process was additionally carried out to obtain *d*_perc,stable_. Data were plotted as the mean ± s.d. of two percolation diameters.

### 2.12. Three-dimensional representation

Volume-rendered models of representative VOIs were generated using *CTVox* software (Bruker, Belgium).

### 2.13. Statistical analysis

Statistical analysis of ‘real’ data was carried out in Graph Pad Prism 7.04. Statistical significance was determined with a one-way ANOVA, followed by Tukey’s HSD with a significance level of *p* = 0.05, since data satisfied the Shapiro–Wilk test for normality.

## 3. Results

### 3.1. Analysis of artificial datasets

#### 3.1.1. Pore size

**Figure 7.**
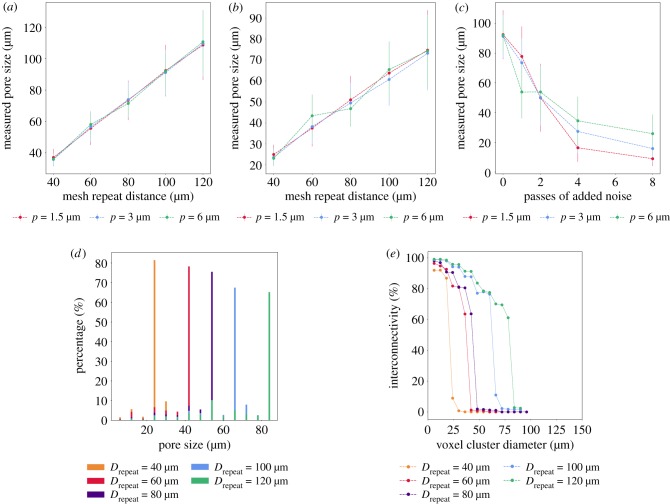
Structural characterization of the artificial datasets: mean pore diameter of the (*a*) aligned (*b*) shifted (*c*) noise-incorporated meshes. Representative distributions of (*d*) pore size distributions and (*e*) interconnectivity obtained for the shifted meshes at a 3 μm simulated pixel size. Here, passes of noise refers to the number of passes the ‘salt and pepper’ function was applied to add noise as defined in §2.2. Error bars represent the standard deviation.

Pore sizes were measured for each mesh repeat distance in the aligned and shifted lattice. As seen in figure 7*a*,*b*, linear regression of the pore sizes revealed aligned meshes to have an average pore size approximately 0.9 times the mesh repeat distance, averaged across the three pixel sizes. Shifted lattices, on the other hand, were roughly 1.6 times smaller than the mesh repeat distances. Considering noise incorporation to aligned datasets with a mesh repeat distance, *D*_repeat_ = 100 μm. Figure 7*c* reveals a decrease in mean pore size at increasing passes of ‘salt and pepper’ noise incorporation. The discrepancies between the pixel sizes are greatest at the highest levels of noise incorporation, with the 6 μm pixel size producing larger mean pore sizes, followed by 3 μm and 1.5 μm.

A narrow distribution of pore sizes is obtained with the aligned meshes as seen in figure 7*d* with the mode coinciding more closely with the mesh repeat distances than the measured pore sizes as observed in figure 7*a* for the aligned and shifted lattices. The best matches between the mesh repeat distance and the mode of the pore size distribution are observed at the lowest pixel size of 1.5 μm. The modes of the pore size distribution of the shifted lattices correspond well with the measured average pore sizes as seen in figure 7*b*.

Similarly, a systematic decrease in the mode of the pore size distribution is observed with decreasing mesh repeat distances in figure 7*d*, corresponding well with the average pore size measured. The standard deviations in the pore size distribution did not vary systematically with increasing degrees of noise incorporation at any pixel size.

**Figure 8.**
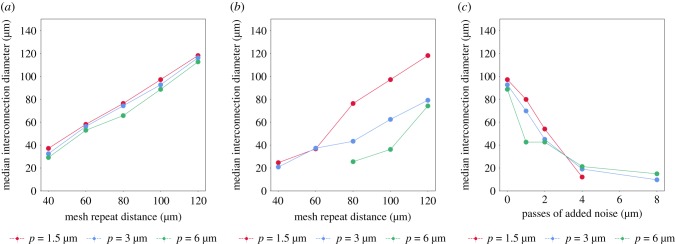
Median interconnection diameter of the (*a*) aligned (*b*) shifted (*c*) noise-incorporated meshes. The median interconnectivity values exhibit trends that are correlated with the average pore size distributions in figure 7*a*–*c*, although the values correlate more strongly with the mode of the pore size distribution rather than the mean values.

A representative interconnectivity plot is shown in figure 7*e* for the shifted lattices simulated at a pixel size of 3 μm, and figure 8 illustrates the extrapolated *median interconnection diameter*, or the value at which 50% interconnectivity is achieved in the structure. The complete set of data pertaining to the pore size distributions and interconnectivity are presented in electronic supplementary material, figures S1 and S2. As expected, the percentage of accessible volume decreased with the mesh repeat distance, and a systematic decrease in the interconnectivity of the scaffold was also observed with increasing degrees of noise as seen in figure 8*c*. The accessible volume did not vary with pixel size, although the ability to compute a median interconnection diameter was impeded at high pixel sizes and low mesh repeat distances. The cluster size at which the sharpest drop-off occurs with these lattices roughly corresponds with the (highest) mode of each pore size distribution observed in figure 7*d*. However, the median interconnection diameters possessed the same values as the mean pore sizes measured in figure 7.

#### 3.1.2. Percolation

**Figure 9.**
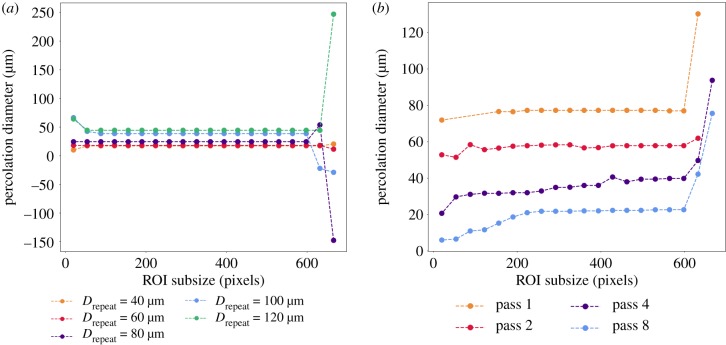
Representative percolation diameters as obtained through the centre ROI subdivision method for (*a*) shifted lattices at mesh repeat distances of 40–120 μm and (*b*) noise incorporated aligned lattices with 1, 2, 4 and 8 passes of the ‘salt and pepper’ function. Datasets analysed here were generated at a simulated pixel size of 3 μm. Percolation diameters observed here demonstrate large variations with subdivision, with regions that can be characterized by a single stable percolation diameter, *d*_perc,stable_.

Representative plots illustrating the percolation diameters obtained with centre subdivision of the ROIs are shown in figure 9. The full set of percolation diameters for the shifted datasets and their associated *R*^2^ values are plotted in electronic supplementary material, figures S3*a* and S4*a* as a function of the subsize in pixels. The percolation diameters displayed variation with subdivision, with regions that can be characterized by a single stable percolation diameter, *d*_perc,stable_. The inclusion of noise to the aligned structures resulted in no negative values of *d*_perc,stable_ observed, through either subdivision methods. However, datasets with increasing degree of noise incorporation exhibited less stability in the percolation diameter with ROI subdivision. In general, the *R*^2^ value increases with subdivision from the largest ROI subsize, for all pixel sizes and subdivision methods used.

Similarly, electronic supplementary material, figures S5*a* and S6*a* represent the percolation diameters obtained through left and centre subdivision of the noise-incorporated datasets. These reveal that negative values of *d*_perc_ were observed at very high or very low subdivision sizes. Percolation diameters were also not obtained consistently at lower subsizes, lower resolution, and at low mesh repeat distances.

The results of the subdivision process can be consolidated into a single table as shown in table 2, where the stability and goodness of fit can be compared for percolation diameters obtained through the different subdivision methods. On the whole, these indicate that all methods are suitable for determining the percolation diameter in a noisy or isotropic structure.

**Table 2.** Summary of the percolation diameters obtained on the artificial datasets using all subdivision methods. All subdivision methods were capable of extracting a positive stable percolation diameter, although the quality of fit decreased slightly with increasing noise (as measured by the *R*^2^ values of the linear fit.)

noise	subdivision	*R*^2^ ranges	sign of *d*_perc,stable_
×	left	>0.9	+ve
×	centre	>0.9	+ve
✓	left	>0.7	+ve
✓	centre	>0.8	+ve

**Figure 10.**
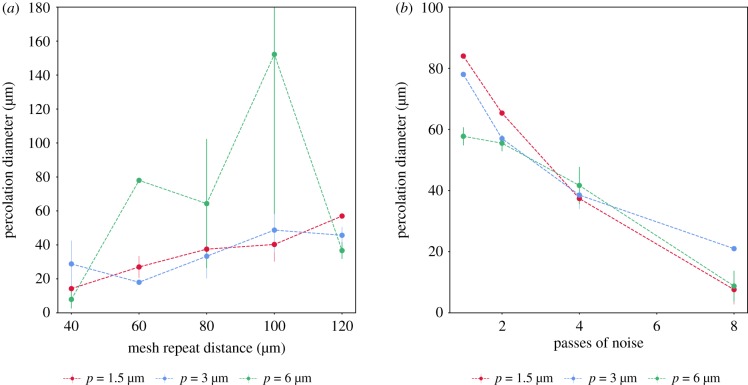
Effective percolation diameter, *d*_perc,stable_ from shifted lattices and noise incorporated lattices using the longest plateau selection criteria. A good match is observed to the mean pore size, particularly for the noise incorporated samples in figure 7*c*. Error bars represent the standard deviation. (*a*) Shifted and (*b*) noise incorporated.

By using the segmented percolation method outlined in §2.6, a single effective percolation diameter, *d*_perc,stable_ was extracted from these datasets and plotted in figure 10*a*,*b*. The *d*_perc,stable_ of the shifted lattices revealed that not all subdivision methods were capable of extricating a percolation for each mesh repeat distance at each pixel size. In general, the values obtained at pixel size 1.5 μm and 3 μm revealed an increase in the percolation diameter with mesh repeat distance. Although a clean trend is not observed, the values were generally consistent with the pore sizes corresponding to the smallest mode of each pore size distribution observed in figure 1.

Similarly, for the datasets with added noise in figure 10*b*, the value of *d*_perc,stable_ demonstrated a strong correlation with the calculated mean pore sizes in figure 7*c*, particularly at 1.5 and 3 μm. At 6 μm, a similar trend is observed and obtained through the left subdivision method.

### 3.2. Analysis of experimental data: structurally variable ice-templated scaffolds

**Figure 11.**
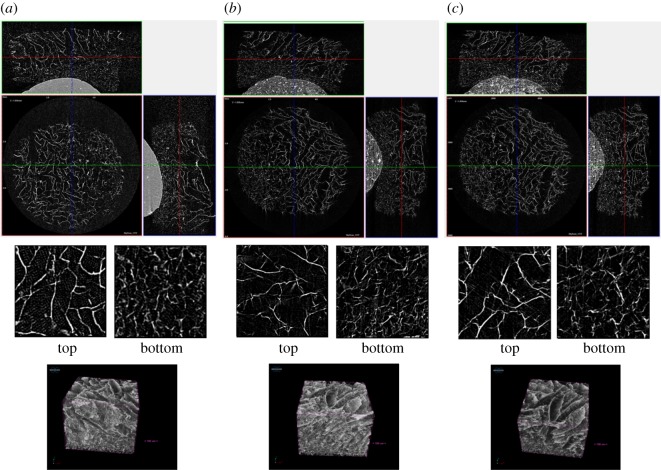
Imaging of the structurally variable scaffolds at the 3 scan pixel sizes. Imaging can be considered at the scale of the whole sample (*a*–*c*) with three orthogonal sections at (*a*) 6 μm pixel size, (*b*) 3 μm pixel size and (*c*) 1.5 μm pixel size. When through-thickness ROIs were considered little appreciable variation was observed in the quality of either individual slices or volume-rendered models. In all instances structural variation between top and bottom regions was observed.

**Figure 12.**
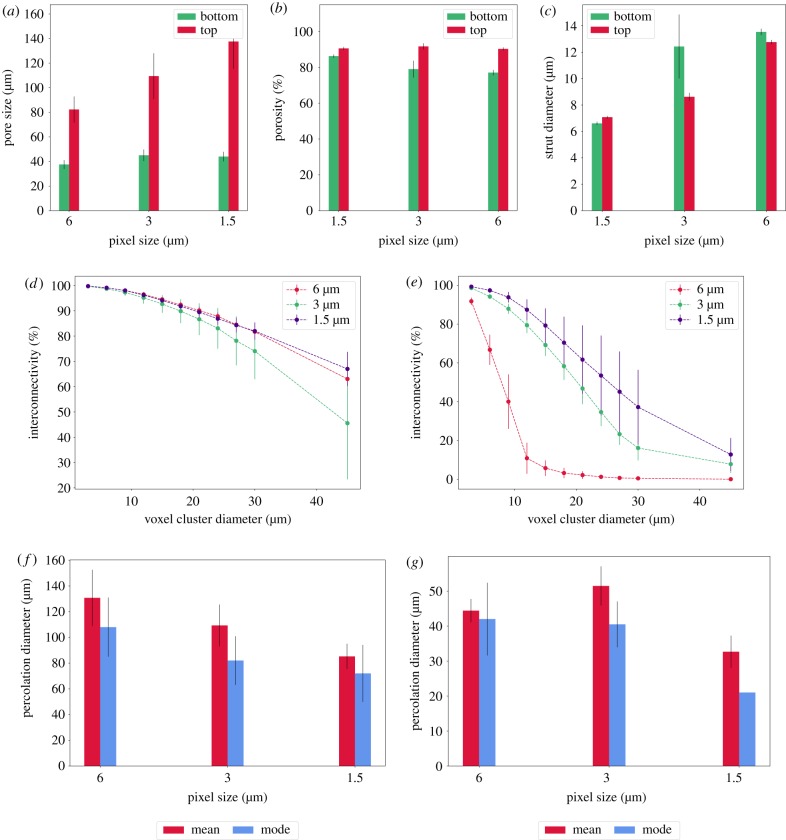
Structural measurements of the dual-layered scaffolds: (*a*) mean pore size, (*b*) volume porosity (*c*) collagen strut thickness, as a function of the pixel size. Volume interconnectivity of the scaffolds are also plotted with respect to interconnection diameter for the (*d*) top and (*e*) bottom sections. Mean and mode pore sizes for the (*f*) top and (*g*) bottom of the structures. Error bars represent the standard deviation.

The cross-sectional images and volume-rendered models of figure 11 demonstrate highly porous interconnected structures with clear structural variation between top and bottom regions. For the purpose of visual imaging alone, there appears little advantage to the longer scan time and higher data use of the lowest resolution scans. However, as shown in figure 12, the structural parameters extracted from the data were affected by the pixel size chosen. There was statistically significant variation between pore size and percentage porosity in the top and bottom layers of the scaffold with all scan resolutions, with a decrease in pixel size from 6 to 1.5 μm resulting in a decrease in the measured mean pore size from approximately 130 μm to around 85 μm. With this decreased mean pore size the associated decrease in interconnectivity with decreasing pixel size was as expected.

### 3.3. Standard percolation analysis

The results of the standard percolation analysis on the real datasets are summarized in table 3. Statistical difference (*p* < 0.05) was observed in percolation diameter between top and bottom regions in all but the 1.5 μm dataset. *R*^2^ values were in all cases highest for the largest pixel size (lowest resolution scan).

**Table 3.** Summary of the percolation analysis on the dual region scaffolds with a percolation diameter, *d*_*c*_ and standard deviations included in parentheses.

section	6 μm		3 μm		1.5 μm	
	*d*_perc_ (μm)	*R*^2^	*d*_perc_ (μm)	*R*^2^	*d*_perc_ (μm)	*R*^2^
top	117.2 (39.1)	0.88 (0.16)	127.4 (31.7)	0.70 (0.22)	88.0 (36.1)	0.56 (0.20)
bottom	0.97 (0.04)	41.3 (6.89)	49.9 (32.5)	0.68 (0.12)	43.0 (16.5)	0.82 (0.11)

#### 3.3.1. Segmented percolation analysis

**Figure 13.**
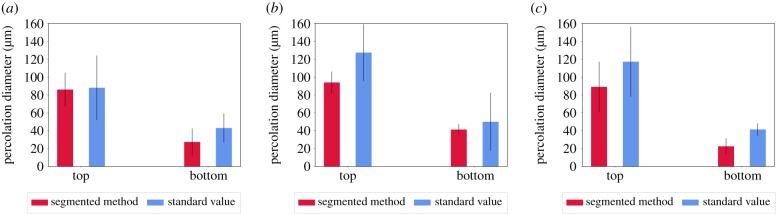
A comparison of the values obtained for the percolation diameter using the standard method and the segmented percolation method at the pixel sizes of (*a*) 1.5 μm, (*b*) 3.0 μm and (*c*) 6 μm. Error bars represent the standard deviation.

A comparison of the values obtained for the percolation diameter using the standard method and the segmented percolation method is presented in figure 13. The values obtained through the standard methods and the segmented percolation method demonstrate similar values for all conditions except the bottom percolation diameter at 6 μm. Trends observed in figure 13 are broadly matched to the means and modes obtained in figure 12, suggesting that the standard method is influenced by the mean pore size whereas the selection criterion for the most stable percolation diameter is influenced by the mode.

## 4. Discussion

### 4.1. Structural measurements

Section 3.1.1 explores the average pore size as well as the pore size distribution of the artificial datasets, and reveals the importance of considering the entire pore size distribution in conjunction with the average pore sizes. Though the standard deviations themselves did not result in appreciable changes to the interconnectivity and percolation results observed, the skew of the distribution (the deviation of the mode from the mean) is speculated to be responsible for the differences observed with the highly anisotropic aligned lattices, when compared with their pore size averages. A theoretical analysis by Tian *et al.* [14] compared the effect of both the average radius and the width of a pore size distribution on the permeability of shale rocks and ultra-filtration membranes. These analyses used the inherently skewed distributions such as the gamma distribution, or log-normal and truncated normal distributions in place of a Gaussian to model the pore size distributions expected in the material. In both materials, an increase in the (non-symmetric) width of the pore size distribution was concluded to increase the permeability of the mineral [14] or the hindered diffusivity of solutes [15] at a constant effective pore radius.

Percolation diameters were not obtained for aligned lattices since extrapolation to the critical cluster size below which an infinite lattice can be fully percolated requires clusters to partially infiltrate the 3D lattice during the shrink-wrap analysis. In spite of the predictions made by the ‘capillary bundle model’ of circular [11,16] cross-sections, since the aligned structures do not have any connections between the walls, all probes of a size smaller than the pore size will fully percolate the structure, whereas all probes larger than this pore size will be unable to access any of the structure.

**Figure 14.**
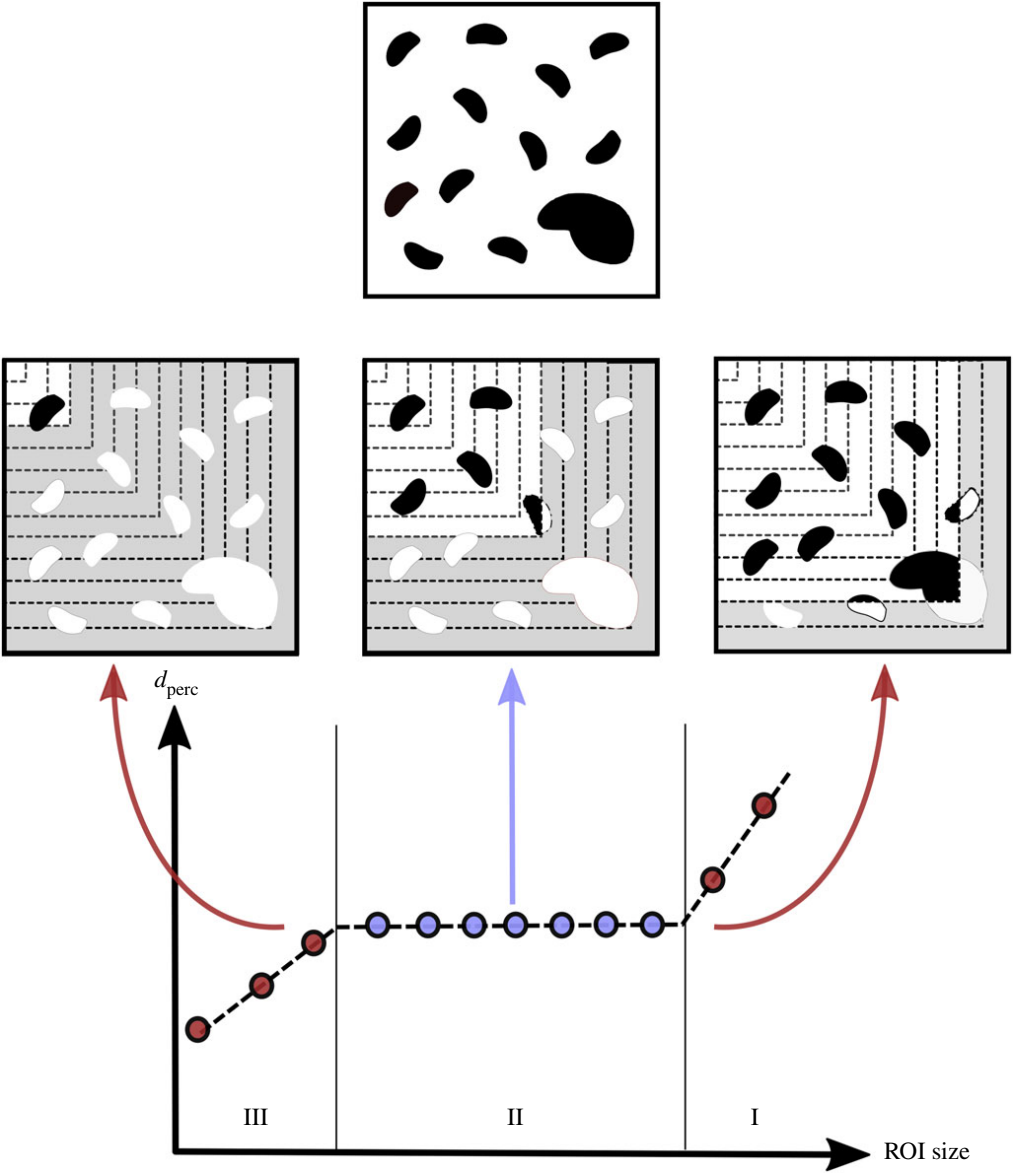
Regions of stability with percolation subdivision. Region I is characterized by a percolation diameter corresponding to the value obtained for the whole dataset. The region is heavily influenced by the presence of any anomalous, often large, pores. Region II represents the region over which the VOI produced a stable percolation diameter as the presence of anomalous features are eliminated. As the VOI becomes smaller than the pore sizes of each lattice, the values observed plummet as seen in Region III.

Figure 14 schematically illustrates an ROI containing anomalous features. Here, two to three distinct regions of percolation diameters are measured as the VOI is reduced in size. The first region (Region I) is characterized by a percolation diameter corresponding to the value obtained for the whole dataset. As the dataset is reduced in size and the influence of any (large) pores on the edge is eliminated, a second stable region (Region II) of a percolation diameter is obtained. For some datasets, such as the 100 μm lattice simulated at 1.5 μm pixel size and the 80 μm lattice simulated at 6 μm pixel size, the percolation diameter also drops drastically as the VOI becomes smaller than the pore sizes of each lattice resulting in the values observed in the final region (Region III).

The issues pertaining to the truncation of the VOI to a size lower than the pore size in Region III are also seen in MicroCT datasets, where the subdivision is not considered. For instance, the idea of a representative volume element underlies the choice of VOI [5]. A representative volume element allows a subset of the entire dataset to be used for percolation analysis to minimize the cost of computation, while possessing the same average structural features of the dataset within the smaller element. The method adopted here to determine convergence of the percolation diameter on average, is not dissimilar to related results from literature where a moving window method was employed to determine the RVE required for percolation analysis [5], as well as the convergence observed in the percolation diameter as the system size [17].

The lack of stability in the percolation diameter observed with both the left and centre subdivision method indicates homogeneity in the lattices as smaller VOIs were obtained by sectioning region towards the top left corner, or the middle of the sample. For the samples analysed in this paper, this suggests that any anomalous pores were not localized at the centre or left corner, and therefore likely to be present along at least one of the other three edges of the VOI. Furthermore, the inability to obtain a positive percolation diameter from the bottom of the stack when using the left ROI subdivision method suggests that both subdivision methods must be applied to ensure that all ‘systematic’ anomalies were accounted for. In particular with the shifted lattices, this behaviour arises from the manner in which the grid was generated. The shift as applied from the top of the stack for these ‘shifted’ lattices occurs from the top left corner to the bottom right corner of the ROI. This effect is inverted when measuring from the bottom of the stack, i.e. the shift of the grid lines appear to move towards the top left corner when moving up from the bottom of the stack. As a result, the left ROI subdivision method would be unable to filter out such anomalies, since these features ‘move’ with the ROI from the bottom right corner to the top left corner as it is reduced in size. Unlike random artefacts that arise from noise during data acquisition or bubbles during scaffold fabrication, examples of systematic anomalies that can occur in real datasets may include fabrication-driven artefacts such the inclusion site of a thermocouple in the slurry or issues during imaging such as the presence of dead camera pixels resulting in ring artefacts during reconstruction.

Additionally, the percolation diameters obtained at the highest ROI size using the centre and left subdivision do not match exactly, since the centre subdivision method requires the identification of the centre of the image. Since the number of pixels is discrete and middle pixel cannot be a half-integer, images with an uneven number of pixels, such as 333 or 1333, will not produce an exact match whereas images with an even pixel width such as 666 will be able to produce consistent results.

The datasets presented here possess a high degree of anisotropy (which is reduced upon noise incorporation), which has been shown to produce negative intercepts with a subset of the subdivision processes. Since the shrink-wrap method involves the inflation and deflation of voxel clusters present in the structure, regular structures such as octahedra and cubes can undergo the process without loss of their aspect ratio [5]. On the other hand, a highly anisotropic pore when represented as a voxel cluster will also transform into an octahedron, resulting in a shift in the overall isotropy of the component [5]. This may be responsible for occurrence of negative values along the anomalous pore channels in Region I, as well as the decreased occurrence of negative percolation diameters upon noise incorporation (and therefore, increase in isotropy).

### 4.2. Determining the ideal pixel size

The inability to obtain percolation diameters at very low or very high pore sizes as seen in figure 10, indicates that significant thought must be given to the choice of pixel size at the data acquisition stage for MicroCT datasets. The determination of the ideal pixel size for percolation analysis suggests that optimization of two different components is required: the maximum pixel size allowable given the smallest feature we must resolve, *p*_res_ and the minimum pixel size that is practical given the computational limits on voxel cluster sizes *p*_comp_. The pixel size must be chosen such that resolution of all salient features is possible with an acceptable computational cost, giving rise to the inequality:

$$p_{\mathrm{comp}}<p_{\mathrm{analysis}}<p_{\mathrm{res}}.$$

#### 4.2.1. Computation

**Figure 15.**
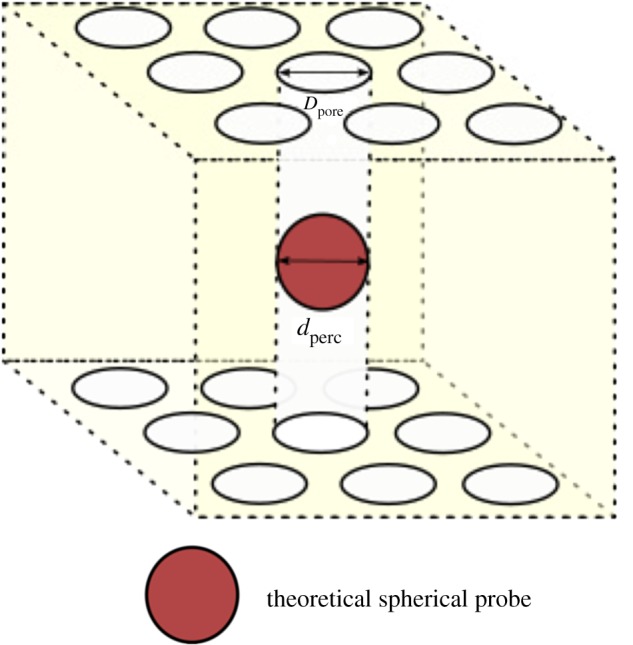
A framework to understand the theoretical limits of percolation diameters. A cuboidal VOI with cylindrical, infinitely extending pathways characterized by a pore size *D*_pore_. The maximum diameter that a penetrating sphere can possess, *d*_perc_ is therefore equal to *D*_pore_.

Considering an ideal, infinitely extending channel of pores, the largest diameter of a sphere that can penetrate the entire network will be given by the pore size, *D*_pore_, as seen in figure 15. This length will, therefore, determine the upper limit of the percolation diameter *d*_perc_.

Due to the high computational cost of running the shrink-wrap algorithm, most analyses are also limited to a finite range of voxel cluster sizes. This implies a minimum voxel cluster size of *c*_min_ and a maximum voxel cluster size of *c*_max_, with the range of encompassing voxel clusters that vary uniformly between *c*_min_ and *c*_max_ with an integer step size—or a granularity—of *g*_*c*_.

**Figure 16.**
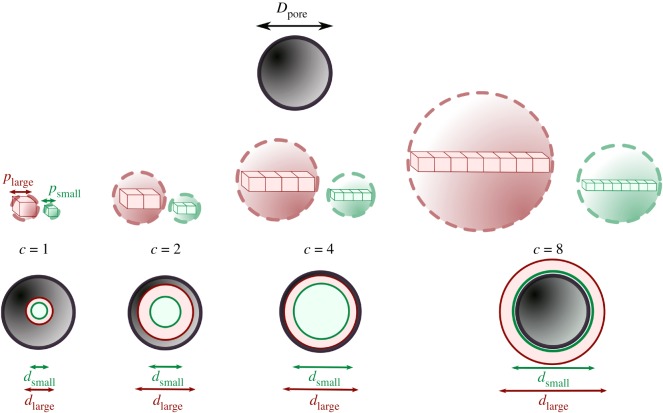
Probing structural elements with voxel clusters at different pixel sizes. A structural element, such as a pore, of size *D*_pore_ can be probed by clusters of various voxel cluster sizes using the shrink-wrap algorithm. In order to obtain a percolation diameter, some voxel clusters must be blocked by the structure, and hence these voxel clusters must be larger than *D*_pore_. The ability to encompass the object entirely is dependent on the pixel size chosen. At *p*_small_, an 8-voxel cluster (green) is required unlike the 4-voxel cluster (red) which can encompass the same feature at *p*_large_.

In order to carry out the percolation analysis successfully, the extrapolation requires the use of voxel clusters with a diameter larger than *d*_perc_. In the example shown in figure 16, a real structural feature of size *D*_pore_, can be fully encompassed by a 4-voxel cluster at a larger pixel size, *p*_large_ (red). The same object is enclosed by a 8-voxel cluster when imaged using a smaller pixel size, *p*_small_ (green).

The highest voxel cluster size, *c*_max_, when dimensionalized using the pixel size, *p*_comp_, must fully encompass the pore and therefore equal *D*_pore_

$$p_{\mathrm{comp}}=\frac{D_{\mathrm{pore}}}{c_{\max}}.$$

However, pore sizes are rarely uniform in reality: the values quoted represent the average in a system with large deviations.

Thus, we must take the largest pore that is expected to be present in the structure, $D_{\mathrm{pore}}^{\max}$ as an upper bound for our maximum computable feature size. Where $D_{\mathrm{pore}}^{\max}$ is not directly known, we can estimate its value given the spread of the individual pore sizes in each dataset. For an expected pore size, *D*_pore_, standard deviation of *σ*_pore_, and a 95% confidence level, we can estimate the maximum pore size to be

$$D_{\mathrm{pore}}^{\max}=D_{\mathrm{pore}}+2\sigma_{\mathrm{pore}}$$

and hence, the minimum pixel size needed to resolve this feature is given by

$$p_{\mathrm{comp}}=\frac{D_{\mathrm{pore}}+2\sigma_{\mathrm{pore}}}{c_{\max}}.$$

#### 4.2.2. Resolution

The resolution of the features detected are not only determined by the pixel size of the scan, *p*, but also the minimum voxel cluster size *c*_min_. Ideally, the pixel size used should be small enough to resolve the smallest possible fenestrations found in the structure of size *D*_fen_ as seen in figure 18. However, with real structures, the smallest of such fenestrations may not be at a practically or physiologically relevant length scale. For instance, the pathways formed by the presence of several openings of *D*_fen_ ∼ 1 nm represent little value to understanding the movement of cell nuclei or drug delivery agents that are of the order of 100–1000 nm, although they may become relevant when characterizing of fluid flow. This minimum feature size to be resolved can instead be set to *d*_object_, or the smallest real incompressible percolating object of interest, for example, a protein molecule or a cell.

For an open, interconnected structure with an average pore size *D*_pore_ > *d*_object_, the minimum resolvable feature size must be sufficiently small to ensure that *c*_min_ is smaller than the features in the VOI. This ensures that voxel cluster probes have sufficient resolution to access the distances at which the partial penetration of the VOI to extrapolate *d*_perc_ is achieved. The minimum value these features can be expected take is expressed as

$$D_{\mathrm{pore}}^{\min}=D_{\mathrm{pore}}-2\sigma_{\mathrm{pore}}$$

and where *D*_fen_ ≈ *d*_object_, the maximum pixel size that is capable of resolving these features is

$$p_{\mathrm{res}}=\frac{D_{\mathrm{fen}}}{c_{\min}}$$

and where *d*_object_ ≫*D*_fen_, the maximum pixel size compatible with resolution is

$$p_{\mathrm{res}}=\frac{D_{\mathrm{pore}}^{\min}}{c_{\min}}.$$

### 4.3. Quantity of data points required for linear regression

**Figure 17.**
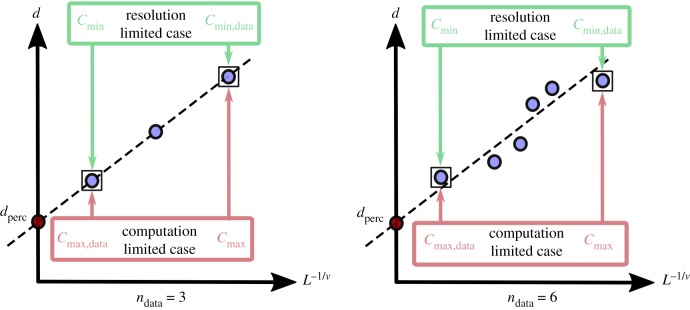
Regression constraints placed by the shrink-wrap analysis to obtain a percolation diameter. The cluster sizes, *d* and corresponding percolation depths *L* plotted can be used to obtain the critical cluster size of percolation *d*_perc_ for an infinitely extending pathway. The number of data points, *n*_data_ required to assure a good linear fit further restricts the clusters that must fully encompass the object of interest from the computational maximum *c*_max_ to *c*_max,data_ if operating at the higher end of computability, or *c*_min_ to *c*_min,data_ at the lower end of resolvability.

In order to obtain the percolation diameter through linear regression, it is necessary to have a sufficient number of accessible data points *n*_data_ as seen in figure 17.

The choice of *n*_data_ may depend on the margin of error determined to be satisfactory for this analysis. This may vary with the presence of sample heterogeneities. In the case where the ability to perform linear regression is computationally limited by *c*_max_, to get sufficient data points on the curve, we must be able to encompass the necessary feature size by a set of *n*_data_ voxel clusters. Therefore, the new limit becomes smaller than *c*_max_ and termed *c*_max,data_. Since the voxel cluster size at which the object must be fully enclosed is now reduced by *n*_data_, and normalized by the granularity of cluster sizes *g*_*c*_, the upper bound on the maximum cluster size is now redefined to

$$c_{\max,\mathrm{data}}=c_{\max}-g_{c}n_{\mathrm{data}}.$$

In the case where resolution is the limiting factor, to get the necessary number of points for linear regression, the smallest features to resolve are reduced by a set of *n*_data_ voxel clusters. Therefore, the new limit becomes larger than *c*_min_ and termed *c*_min,data_. Similarly, as the possible values at the lower range are curtailed to:

$$c_{\min,\mathrm{data}}=c_{\min}+g_{c}n_{\mathrm{data}}.$$

This further curtails the range of pore sizes to

$$\frac{D_{\mathrm{pore}}^{\max}}{c_{\max}-g_{c}n_{\mathrm{data}}}<p<\frac{D_{\mathrm{fen}}}{c_{\min}}$$

or, where *d*_object_ ≫ *D*_fen_

$$\begin{array}{rl} & \frac{D_{\mathrm{pore}}^{\max}}{c_{\max}-g_{c}n_{\mathrm{data}}}<p<\frac{D_{\mathrm{pore}}}{c_{\min}}\\ \;\text{and}\; & \frac{D_{\mathrm{pore}}+2\sigma_{\mathrm{pore}}}{c_{\max}-g_{c}n_{\mathrm{data}}}<p<\frac{D_{\mathrm{pore}}-2\sigma_{\mathrm{pore}}}{c_{\min}+g_{c}n_{\mathrm{data}}}. \end{array}$$

As a worked example, we can apply the above equation to the study of cell percolation in scaffolds modelled as hard spheres. For an estimated pore size of 100 μm, and standard deviation of 20 μm, average mammalian nucleus size of 10 μm, with *c*_min_ = 2, *c*_max_ = 100, *g*_*c*_ = 2 (as required by e.g. the CTAnalyzer software) and a *n*_data_ = 6, the pixel size of choice *p* (in μm) is bounded as

$$\begin{array}{rl} \frac{100+2\times 20}{100-(2\times 6)} & <p<\frac{100-2\times 20}{2+(2\times 6)}\\ \text{and}\;\;\;\;1.6 & <p<4.3. \end{array}$$

Therefore, for this system, we can conclude that a pixel size of 3 μm satisfies both the constraints of resolution and computation for percolation analysis.

However, it must be noted that the physical relevance of this percolation diameter is heavily linked to the phenomena studied. Given the application of hard-sphere percolation of cell nuclei or micro-particles, *d*_object_ is of the order of 10 μm. If *D*_fen_ were to take a value of approximately 50 nm, the pixel size would be required to both be higher than 1.6 μm and lower than 50 nm, suggesting that the analysis would be likely to produce an inaccurate description of the problem under our current computational and imaging constraints. Furthermore, these predictions will not apply cleanly where a more nuanced approach is taken to cell migration and matrix remodelling, or where percolation analysis is used to characterize the establishment of chemical gradients in the scaffold.

Regardless, this inconsistency in the mismatch of *D*_fen_ and *d*_object_ can be resolved by considering the material nature of object. At every pixel size, *p*, the smallest probe that can detect the features in structure, *d*_probe_, is given by the smallest cluster size that can be used in the percolation analysis, *c*_min_:

$$d_{\mathrm{probe}}=pc_{\min}.$$

In the original work by Shepherd *et al.* the structurally variable collagen scaffolds (also analysed here) were used to sieve platelets while retaining megakaryocytes [6]. A preliminary test using micro-particle filtration revealed that 100% of the larger 20 μm micro-particles were retained in the structure, whereas 40 ± 30% of the smaller 10 μm particles were retained. In comparison, 80 ± 15% of the megakaryocytes and 35 ± 30% of the platelets remained embedded in the scaffolds. At the highest resolution of 6 μm, and a *c*_min_ = 2, the smallest probe size we can simulate using percolation analysis, *d*_probe_, has a value of 12 μm. The percolation diameter of 23 ± 9 μm obtained in figure 13 at 6 μm is physically consistent with the ability of 10 μm micro-particles to pass through the structure entirely, while blocking the passage of 20 μm micro-particles. In this case, due to the incompressible nature of the micro-particles, the inability to resolve *D*_fen_ < *d*_object_ does not pose a problem, since any fenestration lower than the size of the object is in practice, impenetrable by the object.

However, as we move towards softer, compressible objects, such as the megakaryocytes and platelets also considered by Shepherd *et al.* [6], the ability of these spherical objects to probe smaller features in the surrounding environment must also be considered. Cells can form filaments and protrusions of various sizes rapidly to probe their surrounding structure and environment. In order for a protrusion to direct cell migration, the protrusion must be stable and semi-persistent rather than transient. For a protrusion to be stable in a cell, there is often a minimum size it must possess.

For instance, in megakaryocytes, such stable protrusions that direct cell migration have been observed at diameters >3 μm [18]. Therefore, if we now consider pixel size, *p* = 3 μm, the minimum probe sizes we can simulate using percolation analysis with a *c*_min_ = 2 corresponds to a *d*_probe_ of 6 μm and similarly, at *p* = 1.5 μm, *d*_probe_ = 3 μm. The corresponding percolation diameter *d*_perc_ at these pixel sizes, as shown in figure 13 was 41 ± 6 μm at 3 μm and 27 ± 15 μm at 1.5 μm. At these two pixel sizes, the smallest probes that can be simulated at a *c*_min_ = 2 are 6 μm and 3 μm, respectively.

Megakaryocytes have been reported to possess a median diameter of 20 μm [19], and should, therefore, be expected to behave similarly to the micro-particles of 20 μm. However, there was a discrepancy observed by Shepherd *et al.* between the near-complete retention of 20 μm micro-particles, and the retention of 80% of megakaryocytes. One possible explanation for this difference may arise from the ability of a real percolating object to sense and respond to features of its own size. The incompressible nature of the micro-particles implies that features smaller than *d*_object_ are impenetrable to them, and as a result, the inability to resolve these features will not affect the prediction of micro-particle filtration.

In the case of the megakaryocytes, the ability of the cell to probe features smaller than the object itself suggests that the resolution of the image must by fine enough to resolve protrusion-sized features. At this finer resolution, the percolation diameter (41 μm) becomes larger than the megakaryocytes themselves (20 μm), giving a potential explanation for the fact that not all megakaryocytes were retained in the scaffold. Consequently, the pixel size *p* = 3 μm is an appropriate choice for this application of megakaryocyte cell sieving, given that it is physically consistent with both the formation of stable protrusions necessary for megakaryocyte migration through a scaffold, and may also explain the subsequent retention of most (but not all) megakaryocytes.

In summary, two considerations must be made. Firstly, the ideal pixel size range for resolution and computation *p*_analysis_ can be calculated. Then, the flow behaviour and materials properties of the percolating object must be considered. For an incompressible real percolating object of size, *d*_object_, the minimum virtual probe size is given by

$$d_{\mathrm{probe}}=d_{\mathrm{object}},$$

whereas for a real percolating object that can change shape, if the size of the smallest protrusions, *d*_protrusion_ can either be calculated using extension ratios and compressibilities, or experimentally measured, the minimum virtual probe size is given by:

$$d_{\mathrm{probe}}=d_{\mathrm{protrusion}}.$$

**Figure 18.**
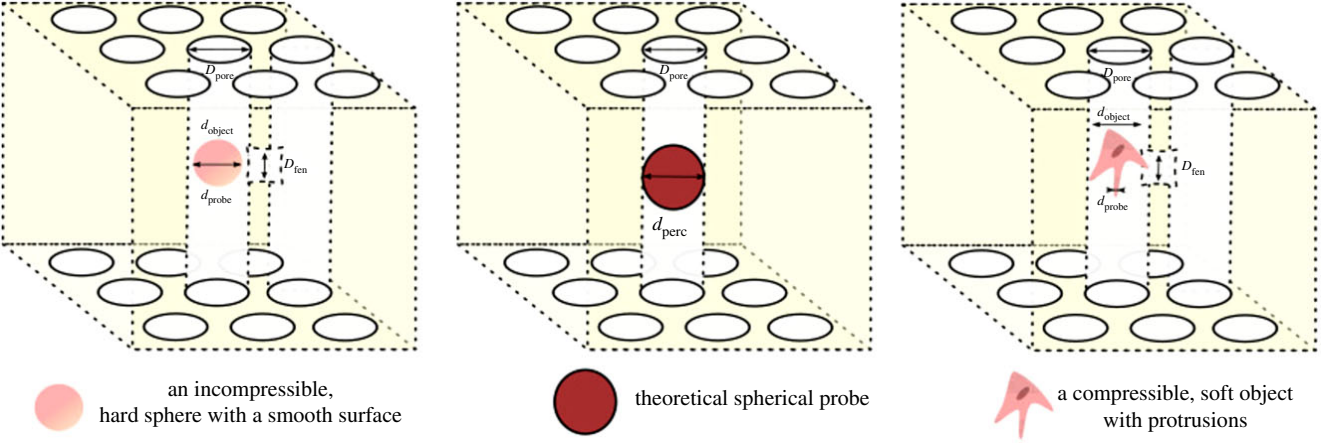
An illustration of the feature sizes of interest when considering the limits of percolation theory. (*a*) A cuboidal VOI of cylindrical, infinitely extending pathways characterized by a pore size *D*_pore_. The maximum diameter that a penetrating sphere can possess, *d*_perc_ is therefore equal to *D*_pore_. (*b*) The cylindrical pathways are now permitted to have uniform fenestrations of size *D*_fen_ that connect the cylindrical pathways. These fenestrations would therefore set a minimum value for the *d*_perc_ to *D*_fen_. However, the inclusion of such fenestrations in calculating *d*_perc_ is insignificant for an object where the minimum probe size on the object *d*_probe_ is significantly larger than *D*_fen_. Thus the material properties of an object of size *d*_object_ will affect the values of *d*_probe_ the object can possess, and as a result, the pathways that can be accessed by the object.

This pixel size, *p*_experiment_, emulating the experimental probe is then given by *d*_protrusion_/*c*_min_ as seen in figure 18. For tissue engineering, although protrusions from the cell are not necessary, they represent one of the most commonly observed features during migration. One of the thinnest protrusions in cells, filopodia, possess a characteristic length between 1 and 2 μm [20] whereas broader lamellipodia responsible for cell migration have been observed at widths between 1 and 5 μm [21]. Consequently, these diameters for lamellipodia or filopodia can be used as *d*_protrusion_ in the general case of mesenchymal cell migration.

If *p*_experiment_ is within the range of values constrained by *p*_analysis_, then *p*_experiment_ can be chosen as the pixel size. In other cases, the structures and materials considered may be unsuitable for percolation analysis without access to more computationally efficient algorithms or bespoke software.

Considering the case of cell migration with lamellipodia, as well as the example MicroCT structures considered above, a pixel size of 3 μm remains suitable for both structural analysis and the experimental system of choice.

## 5. Summary

In this paper, two measures of interconnectivity—the percolation diameter and volume interconnectivity—were evaluated for artificial MicroCT datasets at various simulated pixel sizes. The analysis of these artificial lattices suggested that the mode of the pore size distribution within each lattice may be a more representative estimate than the mean for strongly anisotropic scaffolds, analysis on a large range of structures is required to validate this statement for a general structure. The mode for all datasets exhibits a strong relationship with the volume interconnectivity and percolation diameters obtained. Finally, the prevalence of negative percolation diameters occurring exclusively for highly anisotropic structures also indicates that values obtained may not be valid for such structures.

Crucially, the subdivision algorithm developed in this paper can aid in obtaining a percolation diameter from datasets where anomalous features may otherwise hinder its extraction. The accuracy of the subdivision algorithm, however, is still conditional on the use of an appropriate pixel size to capture the features of interest. By taking into account the limits of computation and resolution, the ideal pixel size for such analyses was determined to be 3 μm for tissue engineering scaffolds.

With the newfound ability to extract a percolation diameter using the subdivision method, another layer of complexity can now be added to the creation of collagen scaffolds of varied scaffold architecture, including the determination of a physically relevant value.

## Supplementary Material

Electronic Supplementary File

## Data accessibility

Data are available from the University of Cambridge Apollo Open Data Repository at https://doi.org/10.17863/CAM.45740
