# Supplementary material for: MicroCT analysis of connectivity in porous structures: optimizing data acquisition and analytical methods in the context of tissue engineering
Source: J R Soc Interface. 2020 Apr 22;17(165):20190833. doi: 10.1098/rsif.2019.0833 (PMC7211477; doi:10.1098/rsif.2019.0833)
Supplement: Electronic Supplementary File [file rsif20190833supp1.pdf]

Supplementary: MicroCT analysis of connectivity in porous structures: optimising data acquisition and analytical methods in the context of tissue-engineering

1. Pore size distribution of artificial datasets

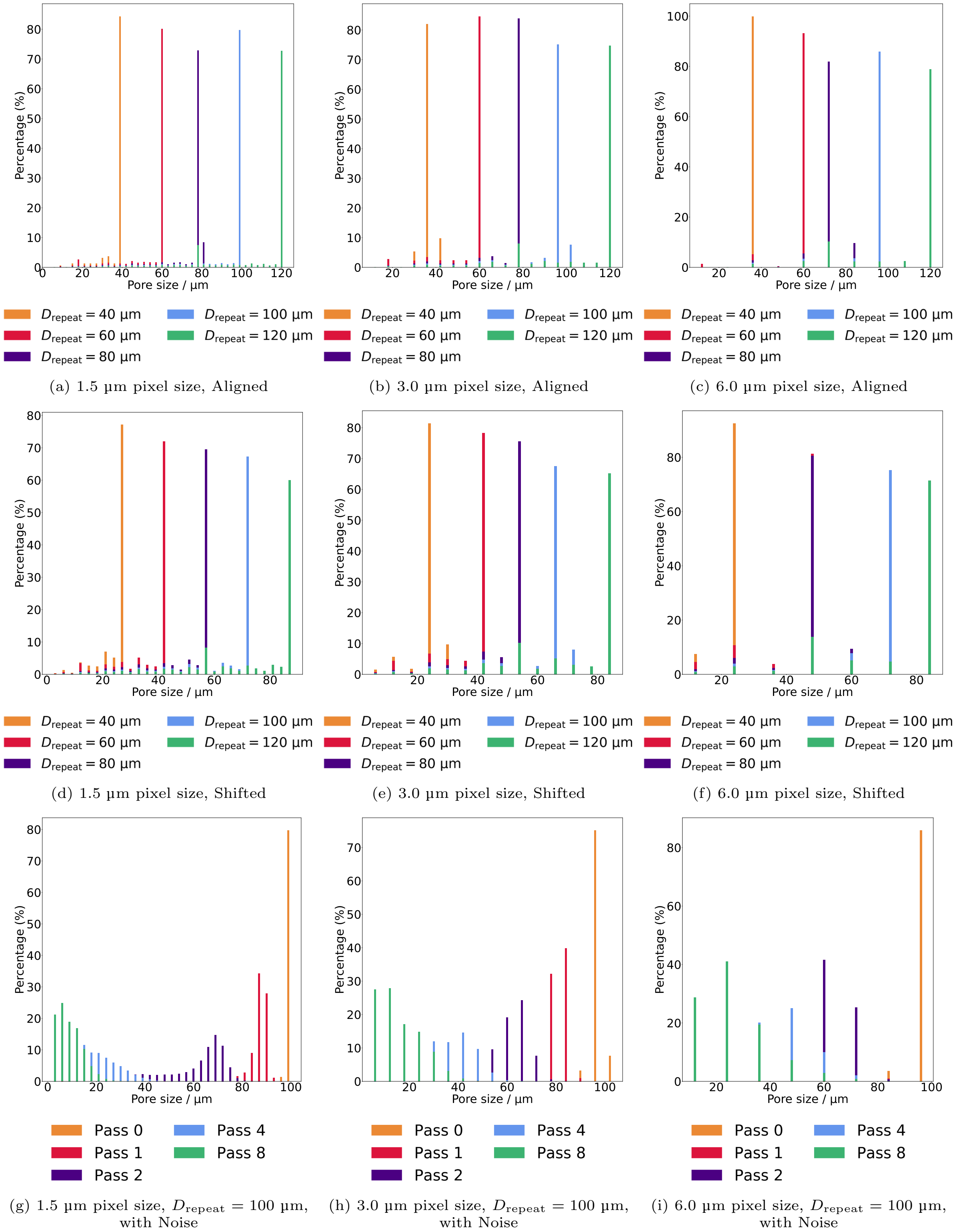

(a) 1.5 µm pixel size, Aligned

(b) 3.0 µm pixel size, Aligned

(c) 6.0 µm pixel size, Aligned

(d) 1.5 µm pixel size, Shifted

(e) 3.0 µm pixel size, Shifted

(f) 6.0 µm pixel size, Shifted

(g) 1.5 µm pixel size, $D_{\mathrm{repeat}} = 100$ µm, with Noise

(h) 3.0 µm pixel size, $D_{\mathrm{repeat}} = 100$ µm, with Noise

(i) 6.0 µm pixel size, $D_{\mathrm{repeat}} = 100$ µm, with Noise

Figure 1: Pore size distribution for aligned, shifted and noisy lattices at different simulated pixel sizes and simulated pore sizes.

2. Interconnectivity of artificial datasets

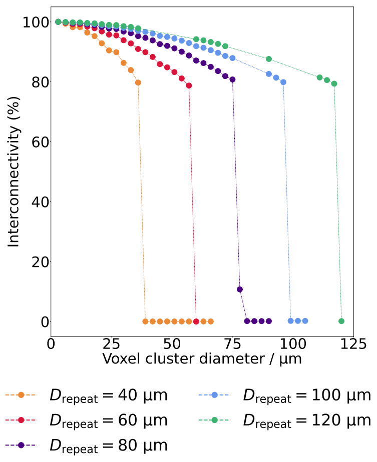

(a) 1.5 µm pixel size, Aligned

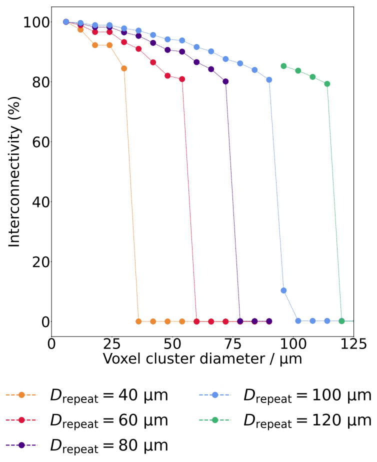

(b) 3.0 µm pixel size, Aligned

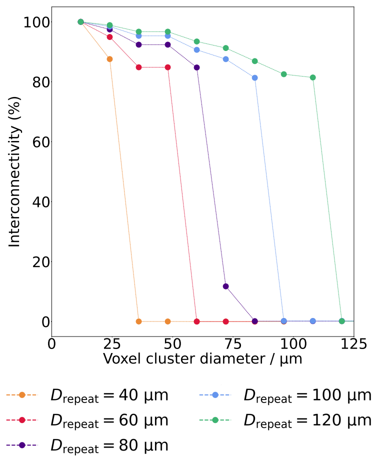

(c) 6.0 µm pixel size, Aligned

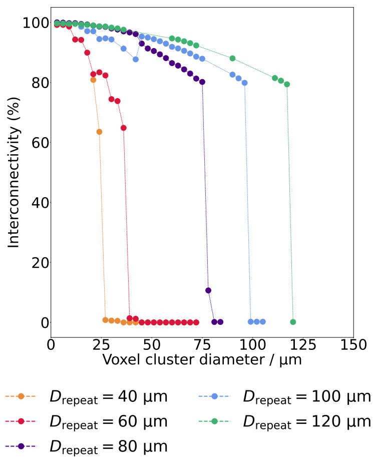

(d) 1.5 µm pixel size, Shifted

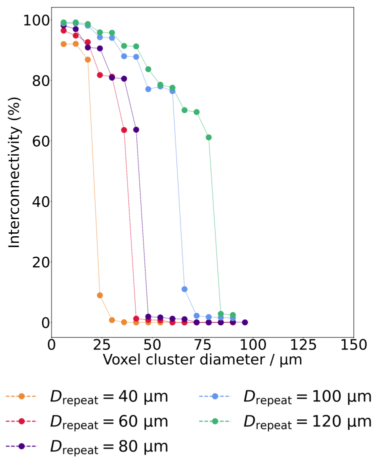

(e) 3.0 µm pixel size, Shifted

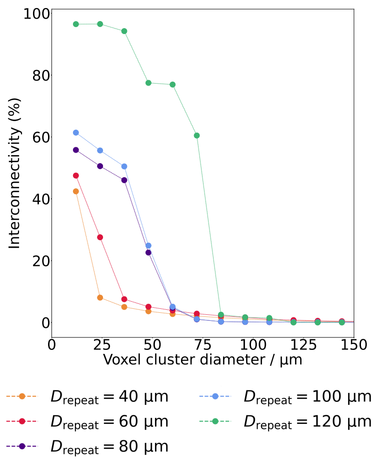

(f) 6.0 µm pixel size, Shifted

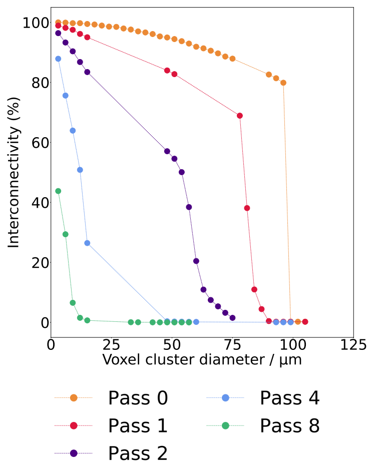

(g) 1.5 µm pixel size, D_{repeat} = 100 µm, with Noise

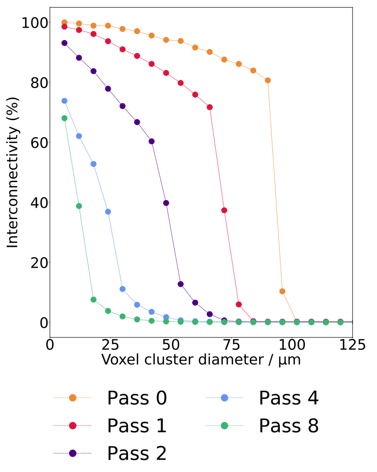

(h) 3.0 µm pixel size, D_{repeat} = 100 µm, with Noise

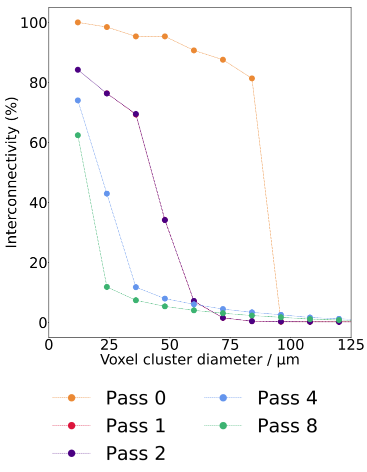

(i) 6.0 µm pixel size, D_{repeat} = 100 µm, with Noise

Figure 2: Volume interconnectivity for aligned, shifted and noisy lattices at different simulated pixel sizes and simulated pore sizes.

3. Percolation diameters of artificial datasets

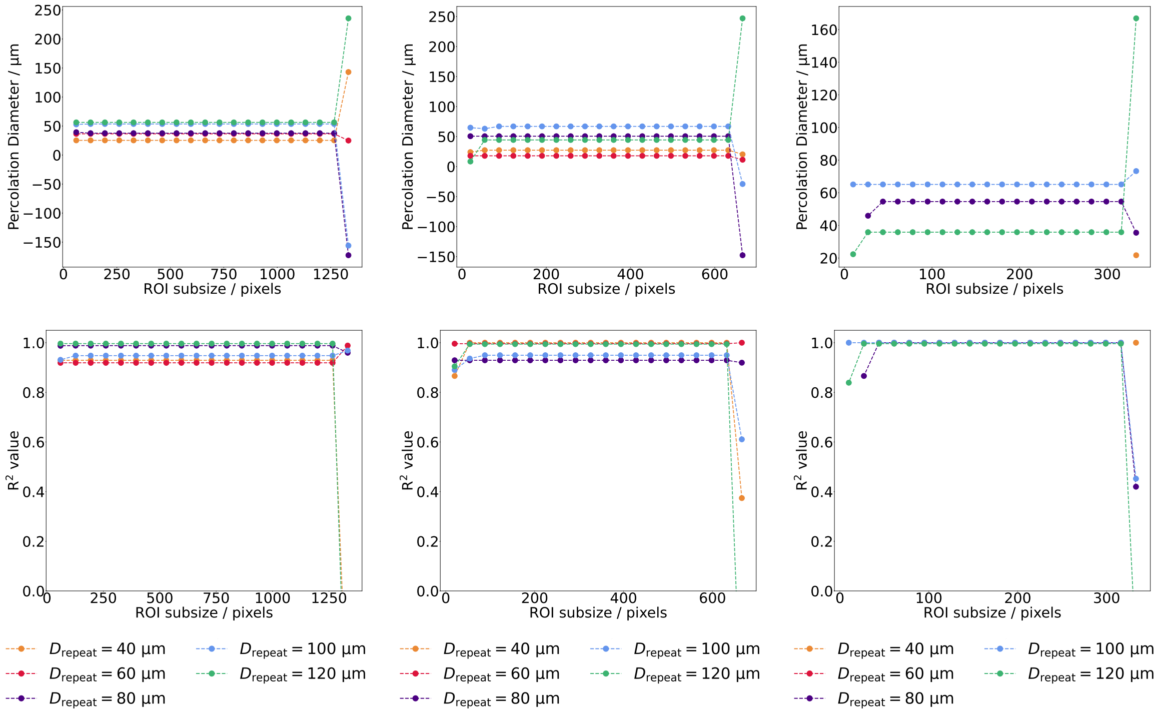

(i) 1.5 µm pixel size (ii) 3.0 µm pixel size (iii) 6.0 µm pixel size

(a) Top percolation diameter

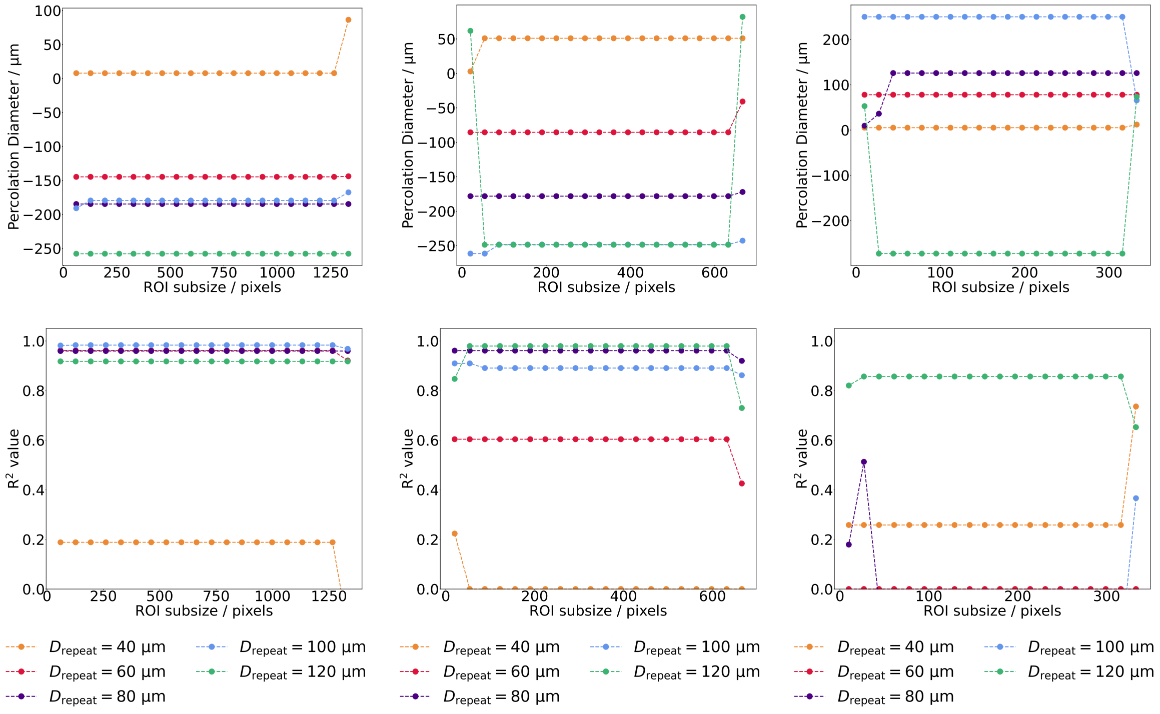

(i) 1.5 µm pixel size (ii) 3.0 µm pixel size (iii) 6.0 µm pixel size

(b) Bottom percolation diameter

Figure 3: Percolation diameter of shifted datasets determined as a function of ROI subsize using the left subdivision algorithm.

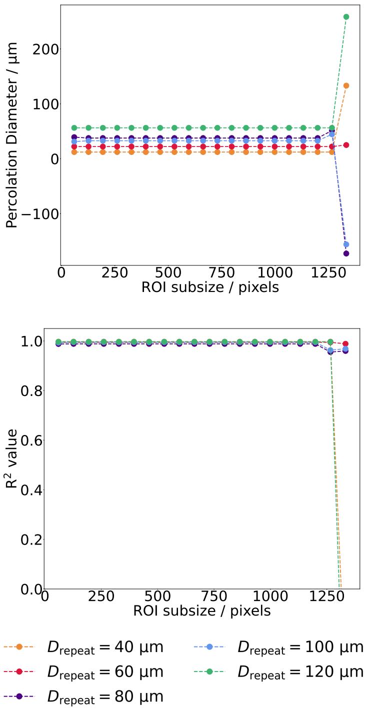

(i) 1.5 µm pixel size

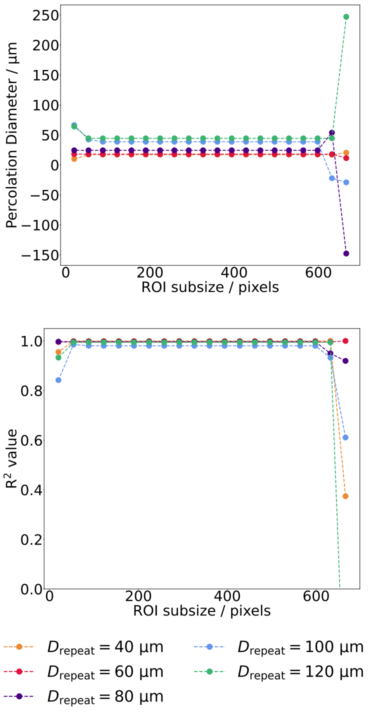

(ii) 3.0 µm pixel size

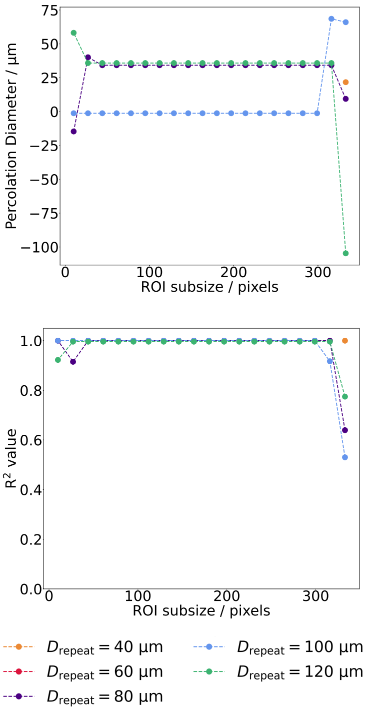

(iii) 6.0 µm pixel size

(a) Top percolation diameter

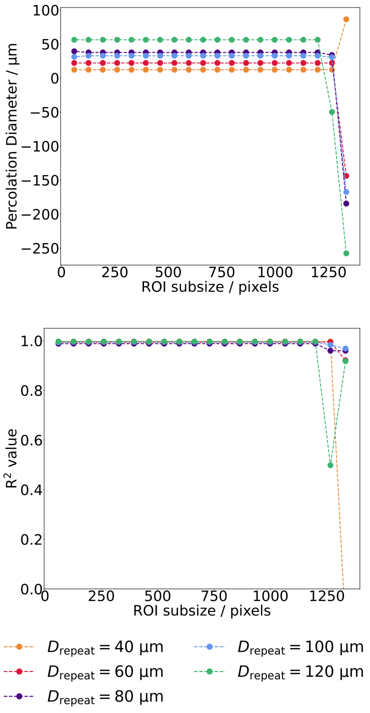

(i) 1.5 µm pixel size

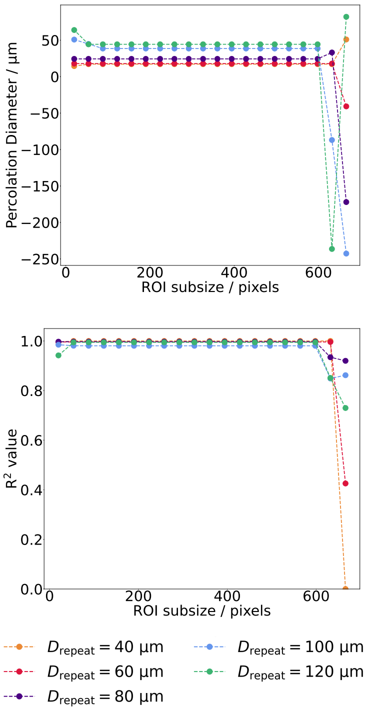

(ii) 3.0 µm pixel size

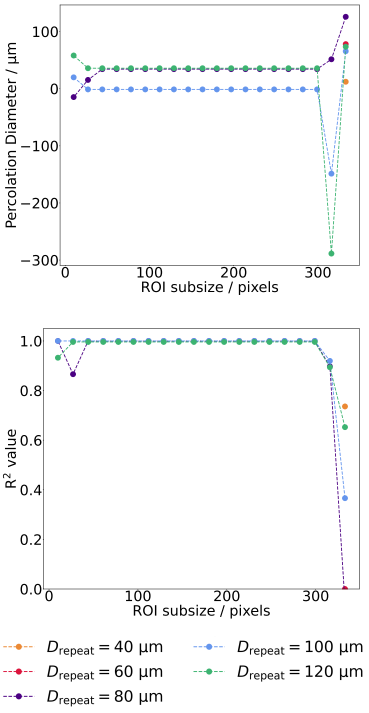

(iii) 6.0 µm pixel size

(b) Bottom percolation diameter

Figure 4: Percolation diameter of shifted datasets determined as a function of ROI subsize using the centre subdivision algorithm.

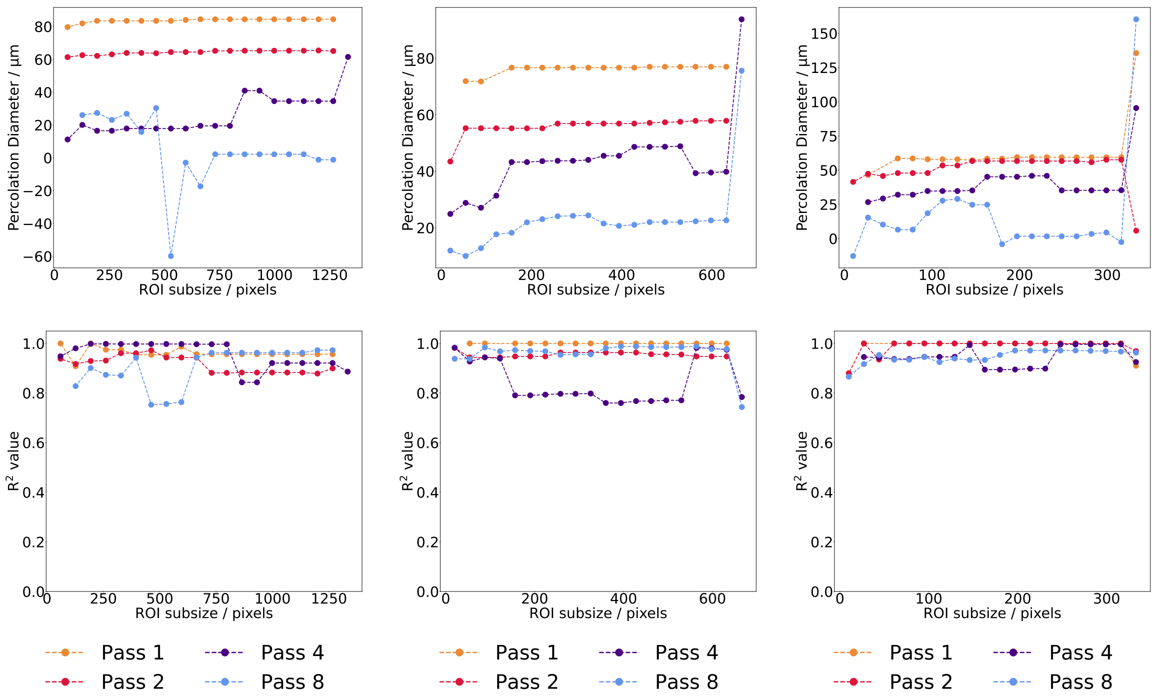

(i) 1.5 µm pixel size (ii) 3.0 µm pixel size (iii) 6.0 µm pixel size

(a) Top percolation diameter

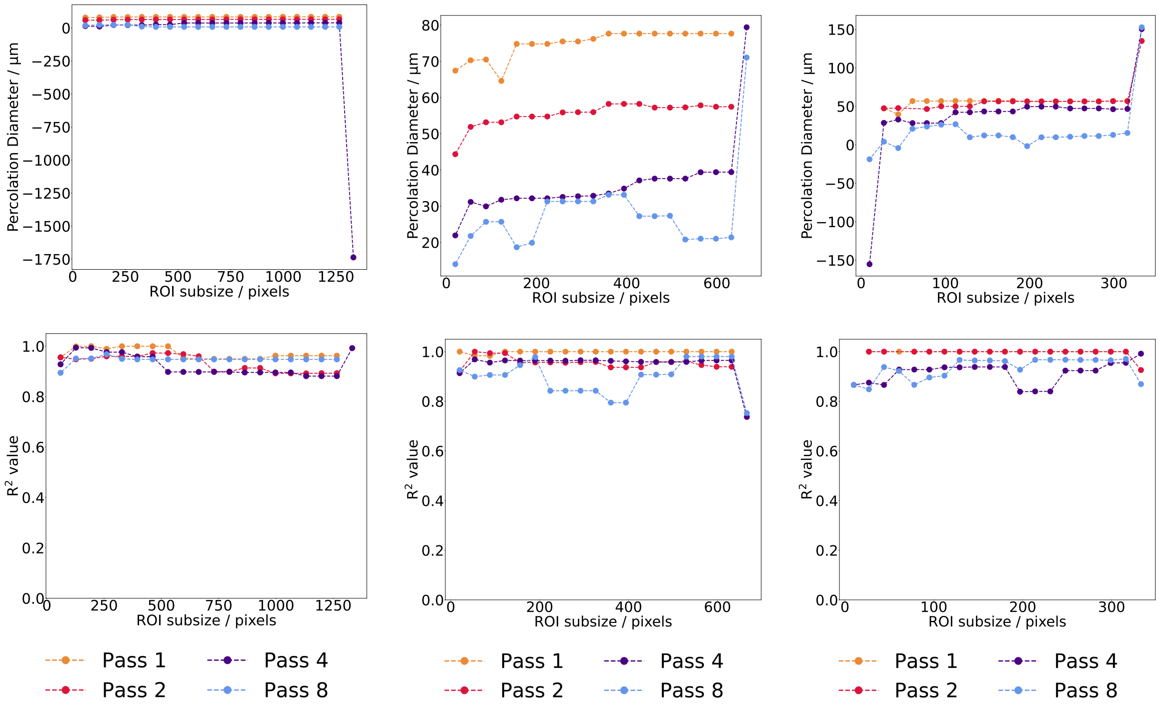

(i) 1.5 µm pixel size (ii) 3.0 µm pixel size (iii) 6.0 µm pixel size

(b) Bottom percolation diameter

Figure 5: Percolation diameter of datasets with added passes of noise determined as a function of ROI subsize using the left subdivision algorithm.

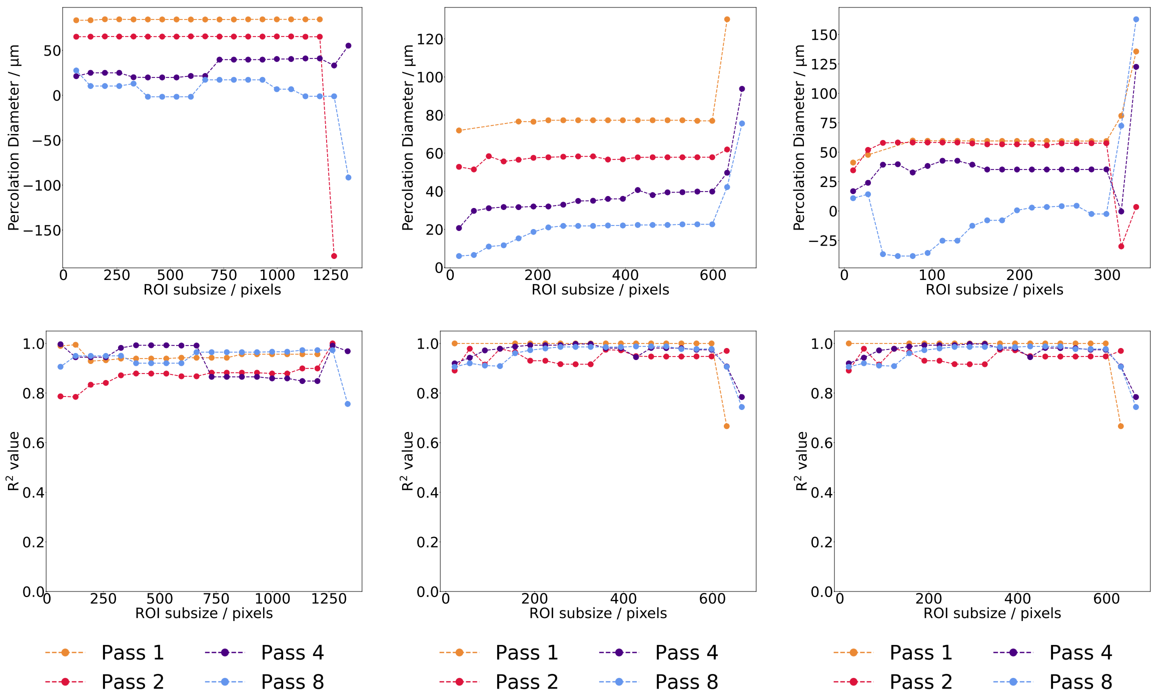

(i) 1.5 µm pixel size (ii) 3.0 µm pixel size (iii) 6.0 µm pixel size

(a) Top percolation diameter

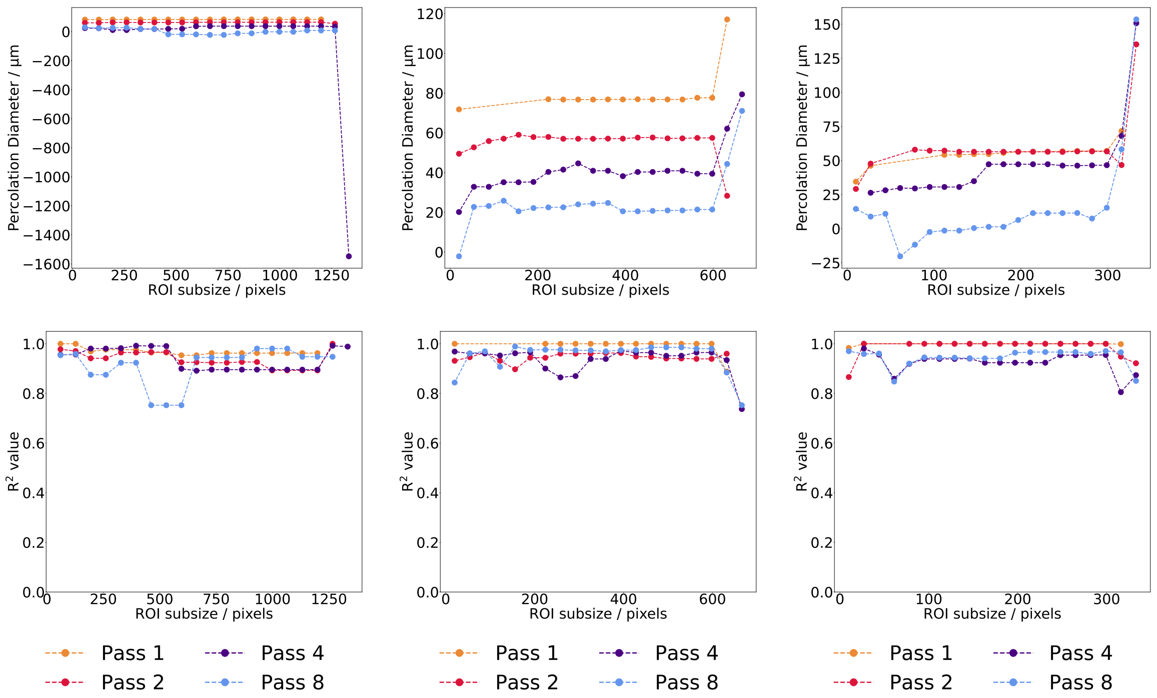

(i) 1.5 µm pixel size (ii) 3.0 µm pixel size (iii) 6.0 µm pixel size

(b) Bottom percolation diameter

Figure 6: Percolation diameter of datasets with added passes of noise determined as a function of ROI subsize using the centre subdivision algorithm.

4. Pore size distribution of real datasets

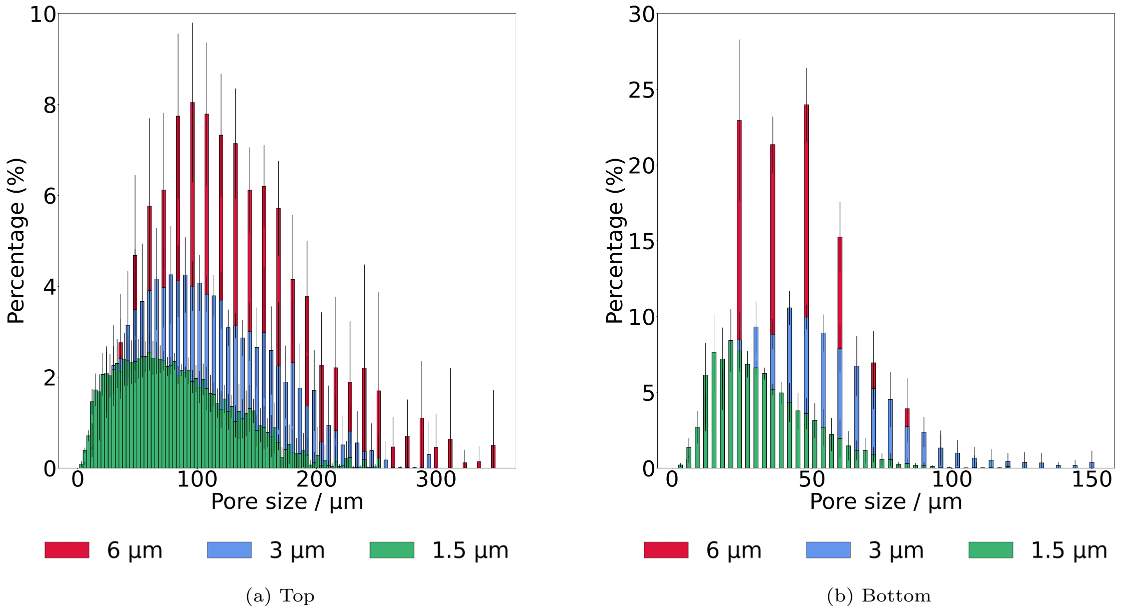

Figure 7: Pore size distributions
